# Kinetic parameters of human aspartate/asparagine–β-hydroxylase suggest that it has a possible function in oxygen sensing

**DOI:** 10.1074/jbc.RA119.012202

**Published:** 2020-02-26

**Authors:** Lennart Brewitz, Anthony Tumber, Christopher J. Schofield

**Affiliations:** Chemistry Research Laboratory, University of Oxford, OX1 3TA Oxford, United Kingdom

**Keywords:** epigenetics, enzyme kinetics, hypoxia, mass spectrometry (MS), hypoxia-inducible factor (HIF), 2-oxoglutarate/alpha-ketoglutarate/2OG-dependent dioxygenase, aspartate/asparagine–beta-hydroxylase/AspH/BAH/HAAH, enzyme assays, epidermal growth factor–like domain, epidermal growth factor–like protein 7/EGFL7

## Abstract

Human aspartate/asparagine–β-hydroxylase (AspH) is a 2-oxoglutarate (2OG)–dependent oxygenase that catalyzes the post-translational hydroxylation of Asp and Asn residues in epidermal growth factor–like domains (EGFDs). Despite its biomedical significance, studies on AspH have long been limited by a lack of assays for its isolated form. Recent structural work has revealed that AspH accepts substrates with a noncanonical EGFD disulfide connectivity (*i.e.* the Cys 1–2, 3–4, 5–6 disulfide pattern). We developed stable cyclic thioether analogues of the noncanonical EGFD AspH substrates to avoid disulfide shuffling. We monitored their hydroxylation by solid-phase extraction coupled to MS. The extent of recombinant AspH-catalyzed cyclic peptide hydroxylation appears to reflect levels of EGFD hydroxylation observed *in vivo*, which vary considerably. We applied the assay to determine the kinetic parameters of human AspH with respect to 2OG, Fe(II), l-ascorbic acid, and substrate and found that these parameters are in the typical ranges for 2OG oxygenases. Of note, a relatively high *K_m_* for O_2_ suggested that O_2_ availability may regulate AspH activity in a biologically relevant manner. We anticipate that the assay will enable the development of selective small-molecule inhibitors for AspH and other human 2OG oxygenases.

## Introduction

**Figure 1.**
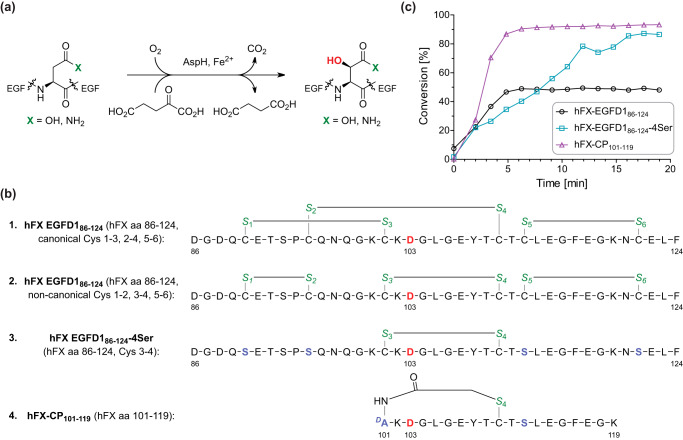
**AspH-catalyzed hydroxylation.** *a*, scheme for the diastereospecific AspH-catalyzed hydroxylation of Asp/Asn residues in EGFDs. *b*, structures of the canonical (*peptide 1*; Cys 1–3, 2–4, 5–6) and noncanonical (*peptide 2*; Cys 1–2, 3–4, 5–6) disulfide isomers of hFX–EGFD1_86–124_, hFX–EGFD1_86–124_-4Ser (*peptide 3*; Cys 3–4), and the cyclic thioether peptide hFX–CP_101–119_ (*peptide 4*). The AspH-hydroxylation site (Asp103_hFX_) is in *red*, cysteine sulfurs are in *green*; and substituted residues are in *light blue*. Numbering is according to the sequence of hFX. *c*, His_6_–AspH_315–758_ catalyzed Asp hydroxylation of hFX–EGFD1_86–124_ (*black circles*, mixture of canonical and noncanonical disulfide isomers, *peptides 1* and *2* in Fig. 1*b*), hFX–EGFD1_86–124_–4Ser (*peptide 3*, *turquoise squares*), and cyclic peptide hFX–CP_101–119_ (*peptide 4*, *purple triangles*) in 50 mm HEPES buffer (pH 7.5, 20 °C). The reactions were performed as described under “Experimental procedures” using 0.1 μm His_6_–AspH_315–758_, 2.0 μm peptide substrate, 100 μm LAA, 10 μm FAS, and 10 μm 2OG. The measurement times were normalized to the first sample injection analyzed after the addition of the enzyme mixture to the substrate mixture (*t* = 0 min), by which time low levels of hydroxylation were manifest.

The human 2-oxoglutarate (2OG^2^, α-ketoglutarate)–dependent aspartate/asparagine–β-hydroxylase (AspH, also called BAH or HAAH) catalyzes the hydroxylation of Asp and Asn residues in epidermal growth factor–like domains (EGFDs) in the endoplasmic reticulum. AspH employs Fe(II) as a co-factor, 2OG and O_2_ as co-substrates producing succinate, and CO_2_ as co-products (Fig. 1*a*) (1, 2). EGFDs of, *inter alia*, coagulation factors and extracellular matrix components such as notch and its ligands, fibrillins, and latent transforming growth factor-β–binding proteins bear the proposed consensus sequence for AspH-catalyzed Asp/Asn hydroxylation (3–5). However, the presence of a consensus sequence alone is not predictive of the extent to which EGFD Asp/Asn hydroxylation occurs *in vivo*. For example, Asp_103_ of human coagulation factor X (hFX) is reported to be hydroxylated quantitatively (6, 7), whereas Asp_123_ of hFVII is reported not to be hydroxylated (8). The factors that regulate the extent of EGFD Asp/Asn hydroxylation and the biochemical consequences of EGFD Asp/Asn hydroxylation are thus poorly understood.

Clinically observed mutations (*e.g.* as occur in the Traboulsi syndrome) in the AspH gene, likely resulting in the loss of oxygenase function, are associated with ophthalmologic defects (ectopia lentis) and facial dysmorphism (9–11). Animal model studies link AspH loss to developmental defects, potentially triggered by disrupted notch signaling (12). AspH levels are up-regulated in certain cancers, *e.g.* hepatocellular carcinoma and glioma (13, 14). One form of AspH is reported to be translocated to the tumor cell surface membrane, an observation correlating with enhanced cell motility and metastatic spread, and statistically reduced life expectancy of cancer patients (15–18). It is unknown how exactly AspH affects cell motility; the biochemical mechanisms, AspH interactions, and substrates underlying this phenotype are not identified, although an effect on notch signaling pathway is proposed (19–21).

More than 100 different human EGFD-containing proteins bear the apparent consensus sequence for Asp/Asn hydroxylation; some of these potential substrates are structurally complex and occasionally contain more than 30 EGFDs (4, 22). Simplified model systems are thus needed to inform on the molecular aspects of AspH biology. AspH activity assays could be applied to determine kinetic parameters of isolated AspH, analyze its substrate affinities, identify inhibitors, investigate how AspH activity is regulated by co-factor/co-substrate availability, investigate the factors determining the extent of *in vivo* EGFD Asp/Asn hydroxylation, and help unravel the mechanisms by which AspH controls cell motility. Such studies, however, have long been limited by the lack of robust assays for isolated AspH.

2OG oxygenases play a pivotal role in the hypoxic response by catalyzing the post-translational prolyl residue hydroxylation of the hypoxia-inducible transcription factors (HIFs) that work to ameliorate the effects of limited oxygen availability (hypoxia) (23, 24). 2OG-dependent HIF-α prolyl hydroxylase (PHD) activity is limited by oxygen availability. Prolyl hydroxylation signals for HIF-α degradation; hence the PHDs are proposed to act as hypoxia sensors (23–25). A second type of HIF-α hydroxylase, factor-inhibiting HIF (FIH), catalyzes HIF-α asparaginyl residue hydroxylation, a modification that serves to reduce the transcriptional activity of HIF (23–25). The HIF-α prolyl and asparaginyl residue hydroxylases contain the typical H*X*(D/E) … H triad of Fe(II)-binding ligands present in most 2OG oxygenases (25). The O_2_-sensing ability of the PHDs is proposed to be reflected in their slow reaction with O_2_, as manifested in high *K_m_* and low *k*_cat_ values (26–29). By contrast, FIH is less susceptible to hypoxia (27, 30–32) and catalyzes the hydroxylation of other substrates than HIF, often from the ankyrin repeat domain–containing family of proteins (33, 34), where it can catalyze the hydroxylation of not only asparaginyl residues, but (like AspH) also of other residues including aspartyl residues (35).

Recently, we described biochemical and crystallographic analyses on AspH (36). The results showed that AspH has an unusual active site, bearing only two Fe(II) ligands (His_679_ and His_725_) rather than the typical H*X*(D/E)…H triad of Fe(II) ligands observed in most 2OG hydroxylases (36). The unusual active site geometry of AspH suggests that it may have the capacity to act as a sensor for Fe(II), 2OG, or O_2_. The combined biochemical and crystallographic studies also revealed that AspH requires a noncanonical EGFD–disulfide connectivity (Cys 1–2, 3–4, 5–6), rather than the canonical EGFD–disulfide connectivity (Cys 1–3, 2–4, 5–6), for productive catalysis (36).

Here, we describe a label-free MS-based AspH activity assay using human His_6_–AspH_315–758_ and synthetic cyclic peptide AspH substrates. The new AspH assay was used to quantify substrate hydroxylation and applied to determine kinetic parameters of AspH for its Fe(II) co-factor, 2OG and O_2_ co-substrates, and stable EGFD substrate analogues. The results suggest that AspH activity has the potential to be limited by O_2_ availability, potentially in a hypoxia-sensing capacity.

## Results

### Development of an efficient AspH activity assay

In our initial report on the activity of AspH, we employed MALDI-MS end-point turnover assays (36). However, these assays required high-enzyme/substrate concentrations and time-consuming sample matrix preparations. This, together with variations in sample ionization efficiencies, disfavors the use of the MALDI-MS assay for efficient high-throughput analyses. Pioneering assays using native AspH had monitored 2OG turnover; however, such assays can be misleading because 2OG oxidation can be decoupled from that of substrate; further, these assays likely involved EGFDs with mixed disulfide patterns (37–40). We therefore aimed to develop improved AspH assays employing defined and stable substrates to investigate the kinetic parameters and substrate selectivity of AspH.

Solid-phase extraction (SPE) coupled to MS was investigated as analytical method to monitor AspH activity. SPE-MS combines the advantages of high resolution MS as a direct label-free technique with the benefits of avoiding time-consuming sample preparation, thus minimizing measurement times. SPE-MS requires only small amounts of substrates/enzymes for analysis and has been successfully applied to monitoring the activity of 2OG oxygenases by measuring mass shifts, *i.e.* +16 Da for hydroxylation and −14 Da for demethylation (41–45). We aimed to combine SPE-MS with the use of stable substrate analogues in which the noncanonical Cys 3–4 EGFD disulfide bond was replaced with a stable thioether.

The initial synthetic substrates used in our study were derived from EGFD1 (amino acids 86–124) of human coagulation factor X (hFX), which is reported to be an AspH substrate in humans (6, 7). The peptides initially studied were hFX–EGFD1_86–124_ (which is a mixture of canonical and noncanonical disulfide isomers; Fig. 1*b*, *peptides 1/2*), hFX–EGFD1_86–124_-4Ser (which has the noncanonical Cys 3–4 EGFD disulfide with the other four cysteine residues substituted for serine residues; Fig. 1*b*, *peptide 3*), and the thioether-linked cyclic peptide hFX–CP_101–119_ (Fig. 1*b*, *peptide 4*). The three substrates all had an aspartyl residue at the hydroxylation site.

The cyclic thioether of hFX–CP_101–119_ (Fig. 1*b*, *peptide 4*) was prepared via reaction of the Cys110_hFX_ thiol with an N-terminal chloroacetyl group and mimics the noncanonical Cys 3–4 EGFD substrate disulfide, while being more stable than a disulfide (36). The three peptides 2, 3, and 4 (Fig. 1*b*) bind to AspH in a catalytically productive manner as evidenced by crystallographic analysis (Fig. S1) (36). The peptides and their hydroxylation products were analyzed by SPE-MS monitoring substrate depletion and product formation (+16 Da mass shift). Initially, the assay conditions were optimized (Fig. S2). The highest AspH activity was observed in 50 mm HEPES buffer (pH 7.5) without additional salts in the presence of 2OG, ferrous ammonium sulfate (FAS), and l-ascorbic acid (LAA, which enhances the activity of many isolated 2OG oxygenases) (Fig. 1*c*) (46–48). Compared with our previously reported MALDI-MS assay conditions (36), the AspH concentration was reduced 100-fold to 0.1 μm in the SPE-MS based assay, significantly reducing the enzyme required, thus potentially more accurately reflecting physiological conditions.

Among the three synthetic AspH substrates investigated, the stable thioether-linked cyclic peptide hFX–CP_101–119_ (Fig. 1*b*, *peptide 4*), which mimics the noncanonical Cys 3–4 hFX EGFD1 disulfide, was most efficiently hydroxylated (Fig. 1*c*). The hydroxylation of hFX–CP_101–119_ and hFX–EGFD1_86–124_ proceeded initially at a comparable rate, implying that the cyclic peptide hFX–CP_101–119_ is a good model system to reflect the hydroxylation of the full-length hFX–EGFD1_86–124_ peptide.

The hFX–EGFD1_86–124_ peptide exists as a mixture of the canonical (Cys 1–3, 2–4, 5–6, the major form; Fig. 1*b*, *peptide 1*) and noncanonical (Cys 1–2, 3–4, 5–6, the minor form; Fig. 1*b*, *peptide 2*) disulfide isomers in solution, of which only the noncanonical isomer is a substrate for AspH (36). Consistent with this, hFX–EGFD1_86–124_ hydroxylation was observed to slow after 5 min, reflecting complete consumption of the active noncanonical disulfide isomer (∼45% conversion; Fig. 1*c*). Although the hydroxylation of hFX–EGFD1_86–124_ can be driven to completion, *i.e.* by adding redox-active tripeptide GSH to the assay enabling disulfide isomerization (36), the presence of such reactive components in the assay is undesirable because they may limit applications such as the profiling of small-molecule AspH inhibitors (GSH might react with redox-active small molecules). Furthermore, disulfide isomerization is relatively slow at 20 °C, complicating kinetic analysis when using the hFX–EGFD1_86–124_ disulfide mixture (Fig. 1*b*, *peptides 1* and *2*) as an AspH substrate.

In an attempt to circumvent the problems caused by hFX–EGFD1_86–124_ disulfide isomerism, AspH-catalyzed hydroxylation of an hFX–EGFD1_86–124_-derivative with only one disulfide, *i.e.* hFX–EGFD1_86–124_-4Ser (Fig. 1*b*, *peptide 3*), was investigated (Fig. 1*c*). However, hydroxylation of hFX–EGFD1_86–124_-4Ser was relatively slow compared with the other substrates, which might reflect a more disordered secondary structure in solution caused by the substitution of four cysteines/two cystine links by serine residues. We therefore pursued further studies using the synthetic thioether-linked cyclic peptide hFX–CP_101–119_ AspH substrate (Fig. 1*b*, *peptide 4*). This has the additional benefit that its synthesis is straightforward and cost-effective.

### Validation of the thioether-linked cyclic peptide as an AspH substrate

The thioether-linked cyclic peptide hFX–CP_101–119_ (Fig. 1*b*, *peptide 4*) was then further validated as a tool to monitor AspH activity. Only a single AspH-catalyzed oxidation event (+16-Da mass shift) was observed under the assay conditions, *i.e.* no overoxidized cyclic peptides (+32 or +48 Da mass shifts) were observed by SPE-MS (Fig. S3). No oxidation of hFX–CP_101–119_ was observed under the assay conditions in the absence of His_6_–AspH_315–758_. Taken together, these experiments show that oxidation of the thioether sulfur atom does not account for the observed mass difference of +16 Da (Fig. S3), in agreement with previous NMR experiments indicating that AspH oxidized Asp_103hFX_ of a thioether-linked cyclic peptide with a shorter sequence (hFX–CP_101–110_) (36).

To investigate whether SPE-MS analysis of hFX–CP_101–119_ can be used to quantify AspH activity, the ion counts of product (hydroxylated) and substrate (nonhydroxylated) cyclic peptides were analyzed as a function of time (Fig. S3); the sum of the ion counts was constant throughout the time course, confirming that SPE-MS is a useful technique to quantify AspH activity using the cyclic peptide hFX–CP_101–119_ as substrate.

**Figure 2.**
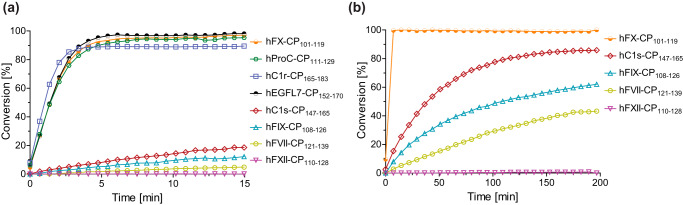
**Time course of the AspH-catalyzed hydroxylation of different thioether-linked cyclic peptides corresponding to reported AspH substrates and controls.** *a*, His_6_–AspH_315–758_ hydroxylates the cyclic peptides hProC–CP_111–129_ (*green*), hC1r–CP_165–183_ (*lavender*), and hEGFL7–CP_152–170_ (*black*) efficiently with complete hydroxylation being observed in less than 15 min in a manner similar to hFX–CP_101–119_ (*orange*). Hydroxylation of hC1s–CP_147–165_ (*red*), hFVII–CP_121–139_ (*yellow*), and hFIX–CP_108–126_ (*cyan*) is considerably slower. No hydroxylation of hFXII–CP_110–128_ (*pink*) was observed. *b*, His_6_–AspH_315–758_ catalyzes the hydroxylation of hFX–CP_101–119_ (*orange*) apparently instantaneously, whereas hFXII–CP_110–128_ (*pink*) is not hydroxylated even when incubated with AspH for 200 min. Hydroxylation of hC1s–CP_147–165_ (*red*), hFVII–CP_121–139_ (*yellow*), and hFIX–CP_108–126_ (*cyan*) proceeds relatively slowly over 200 min. The reactions were performed as described under “Experimental procedures” using 0.1 μm His_6_–AspH_315–758_, 2.0 μm peptide, 100 μm LAA, 10 μm FAS, and 10 μm 2OG in 50 mm HEPES buffer (pH 7.5, 20 °C). The measurement times were normalized to the first sample injection analyzed after the addition of the enzyme mixture to the substrate mixture (*t* = 0 min), by which time low levels of hydroxylation were manifest. Peptide structures are shown in Fig. S4.

To investigate the extent to which hydroxylation of thioether-linked cyclic peptides reflect literature-reported EGFD Asp/Asn-hydroxylation levels in humans, thioether-linked cyclic peptides were synthesized based on the amino acid sequences of reported human AspH substrate proteins other than hFX: human coagulation factor VII (hFVII–CP_121–139_) (8), human coagulation factor IX (hFIX–CP_108–126_) (7), human protein C (hProC–CP_111–129_) (49, 50), human complement C1r subcomponent (hC1r–CP_165–183_) (51, 52), and human complement C1s subcomponent (hC1s–CP_147–165_) (51, 53). These potential substrates include examples with both an aspartyl and an asparaginyl residue at the hydroxylation site. As a potential negative control, a cyclic peptide was synthesized based on the sequence of human coagulation factor XII (hFXII–CP_110–128_), which does not bear the AspH consensus sequence (50). A cyclic peptide based on the sequence of human epidermal growth factor–like protein 7 (hEGFL7–CP_152–170_), which as yet has not been associated with AspH biology, but which bears the predicted AspH substrate consensus sequence, was also synthesized. The relative sequence length (19 amino acids) and secondary structure (position of the thioether linkage) of the cyclic peptides are similar to hFX–CP_101–119_ (Fig. 1*b*, *peptide 4*) as shown in Figs. S1 and S4. The cyclic peptides were incubated with His_6_–AspH_315–758_ under the optimized assay conditions; the extent of their Asp/Asn hydroxylation was monitored using SPE-MS (Fig. 2, Table 1, and Fig. S4).

**Table 1:** **AspH-catalyzed hydroxylation of thioether-linked cyclic peptides based on the EGFD sequences of human proteins**

	Peptide substrate*^a^*	Hydroxylation		Reported *in vivo* EGFD Asp/Asn-hydroxylation levels in humans	
		*t* = 15 min	*t* = 200 min	Values	Sources
		%		%	
1	hFX–CP_101–119_ (Fig. 1*b*, *peptide 4*)	>95	>95	89–100	Refs. 6, 7, and 63
2	hProC–CP_111–129_	>95	ND*^b^*	89–100	Refs. 49 and 50
3	hEGFL7–CP_152–170_	>95	ND		*^c^*
4	hC1r–CP_165–183_	∼90	ND*^d^*	90	Refs. 51 and 52
5	hC1s–CP_147–165_	∼20	∼85	∼50	Refs. 51 and 53
6	hFIX–CP_108–126_	∼12	∼60	26	Ref. 7
7	hFVII–CP_121–139_	∼5	∼40	0	Ref. 8
8	hFXII–CP_110–128_	<1	<1	0	Ref. 50

*^a^* The sequences and structures of the cyclic peptides are shown in Fig. S4.

*^b^* ND, not determined.

*^c^* Note that human EGFL7 is not a reported substrate of AspH.

*^d^* Asn167_hC1r_ hydroxylation did not appear to increase beyond ∼90% substrate conversion.

The results of screening the AspH-catalyzed hydroxylation of the different thioether-linked cyclic peptides are summarized in Table 1, which compares the results with literature-reported *in vivo* hydroxylation levels of the human AspH substrate proteins. The results support previous studies showing that AspH can catalyze efficiently hydroxylation of both Asp and Asn residues. Importantly, the relative extent of cyclic peptide hydroxylation correlates well with the relative levels of both EGFD Asp and Asn hydroxylation observed *in vivo*. For example, hFX–CP_101–119_ is more efficiently hydroxylated by AspH under the assay conditions than hFIX–CP_108–126_, which itself is a better substrate than hFVII–CP_121–139_. The cyclic peptides hProC–CP_111–129_, hC1r–CP_165–183_, and hEGFL7–CP_152–170_ are as efficiently hydroxylated as hFX–CP_101–119_ upon short exposure times to AspH (<15 min), in apparent agreement with the reported *in vivo* EGFD hydroxylation levels for human protein C (49, 50) and C1r (51, 52). The hydroxylation of hFIX–CP_108–126_, hFVII–CP_121–139_, and hC1s–CP_147–165_ was incomplete even after a prolonged exposure time of 200 min (Table 1), again reflecting the reported *in vivo* human EGFD hydroxylation levels (7, 8, 51, 53).

For all the tested cyclic peptides, only one oxidation event (+16-Da mass shift) was observed, with the exception of hFXII–CP_110–128_, which was not a substrate. Together with the divergent hydroxylation levels, this observation further confirms the proposal that AspH selectively catalyzes the anticipated β-oxidation of Asp/Asn residues. No hydroxylation of the thioether-linked cyclic peptide hFXII–CP_110–128_, which contains a methionine residue in its sequence, was observed supporting the proposal that the consensus sequence C*X*(D/N)*XXXX*(F/Y)*X*C for AspH-catalyzed Asp/Asn hydroxylation in EGFDs is valid. The essential consensus sequence hydrophobic Phe/Tyr residue, which interacts with a hydrophobic pocket located in the AspH tetratricopeptide repeat (TPR) domain (Fig. S1) (36), is substituted by a proline in hFXII–CP_110–128_, explaining its lack of activity. Having confirmed that thioether-linked cyclic peptides are useful model systems that apparently reflect *in vivo* AspH-catalyzed human EGFD oxidation, we next focused on determining kinetic parameters for AspH.

### AspH kinetic parameters

The kinetic characterization of His_6_–AspH_315–758_ with respect to its co-factor and (co-)substrates was then performed under the optimized SPE-MS assay conditions using the stable thioether-linked cyclic peptide hFX–CP_101–119_ (Fig. 1*b*, *peptide 4*) as a substrate. To our knowledge, kinetic data of AspH have so far only been reported using the partially purified native bovine-derived AspH and peptides based on the sequence of hFX and hFIX (with undefined disulfide connectivity) as substrates, with monitoring by 2OG turnover (38–40).

**Figure 3.**
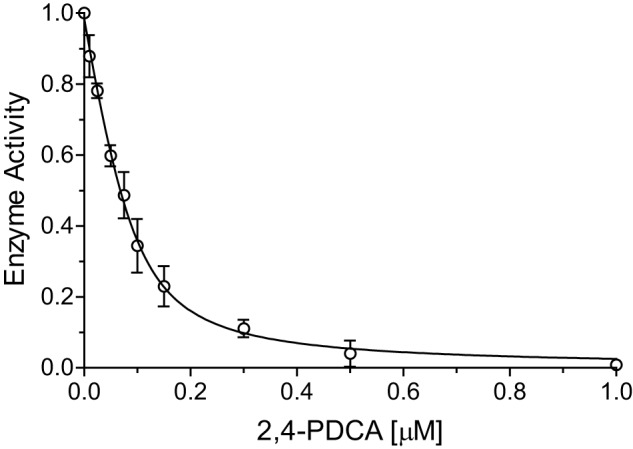
**Active site titration of AspH using pyridine-2,4-dicarboxylic acid (2,4-PDCA) as a tight binding inhibitor.** The data are shown as the mean averages of three independent runs (*n* = 3; means ± S.D.). AspH activity was normalized against a DMSO control. Initial peptide hydroxylation rates are shown in Fig. S5.

Initially, we determined the concentration of active His_6_–AspH_315–758_ by performing an active site titration using the tight binding small-molecule AspH inhibitor pyridine–2,4-dicarboxylic acid (38, 54). Based on the SPE-MS experiments, the total concentration of active His_6_–AspH_315–758_ was calculated to be 90.8 ± 13.7 nm with an original estimated AspH assay concentration of 100 nm (Fig. 3). Based on the calculated concentration of active enzyme, both turnover numbers (catalytic constants, *k*_cat_) and specificity constants (*k*_cat_/*K_m_*) were then determined (see below). Turnover numbers using hFX–CP_101–119_ as an AspH substrate were, within the experimental error, constant throughout all kinetic experiments (Table 2), indicating good data quality and accuracy of the SPE-MS AspH activity assay.

**Table 2:** **Steady-state kinetic parameters for AspH** Maximum velocities (*v*_max_), Michaelis constants (*K_m_*), turnover numbers (*k*_cat_), and specificity constant (*k*_cat_/*K_m_*) of His_6_–AspH_315–758_ for its Fe(II) cofactor, 2OG and O_2_ co-substrates, LAA, and substrates. The values are the mean averages of three independent runs (*n* = 3; means ± S.D.).

		*v*_max_	*K_m_*	*k*_cat_	*k*_cat_/*K_m_*
		μ*m s*^−*1*^	μ*m*	*s*^−*1*^	μ*m*^−*1*^ · *s*^−*1*^
1	Fe(II)*^a^*^,^*^b^*	16.8 × 10^−3^ ± 0.4 × 10^−3^	4.76 ± 0.48	0.19 ± 0.03	0.04 ± 0.01
2	Fe(II) *^a^*^,^*^c^*	17.1 × 10^−3^ ± 0.5 × 10^−3^	1.42 ± 0.16	0.19 ± 0.03	0.13 ± 0.03
3	LAA*^a^*	14.9 × 10^−3^ ± 2.1 × 10^−3^		0.16 ± 0.03	
4	2OG*^a^*	16.8 × 10^−3^ ± 0.5 × 10^−3^	0.60 ± 0.09	0.19 ± 0.03	0.32 ± 0.07
5	hFX–CP_101–119_	17.8 × 10^−3^ ± 1.3 × 10^−3^	1.19 ± 0.26	0.20 ± 0.03	0.17 ± 0.05
6	hProC–CP_111–129_	15.6 × 10^−3^ ± 0.6 × 10^−3^	1.71 ± 0.21	0.17 ± 0.03	0.10 ± 0.02
7	hEGFL7–CP_152–170_	22.8 × 10^−3^ ± 4.3 × 10^−3^	2.04 ± 0.65	0.25 ± 0.06	0.12 ± 0.05
8	hC1r–CP_165–183_	41.0 × 10^−3^ ± 7.0 × 10^−3^	3.00 ± 0.79	0.45 ± 0.10	0.15 ± 0.05
9	hC1s–CP_147–165_	10.9 × 10^−3^ ± 0.6 × 10^−3^	36.5 ± 5.1	0.12 ± 0.02	3.3 × 10^−3^ ± 0.7 × 10^−3^
10	O_2_*^a^*^,^*^d^*	20.7 × 10^−3^ ± 1.8 × 10^−3^	426 ± 73	0.23 ± 0.04	5.4 × 10^−4^ ± 1.3 × 10^−4^

*^a^* The *v*_max_^app^ and *K*_*m*_^app^ values were determined monitoring the hydroxylation of hFX–CP_101–119_ (Fig. 1*b*, *peptide 4*).

*^b^* In the absence of LAA.

*^c^* In the presence of LAA.

*^d^* Mean averages of four independent runs (*n* = 4; means ± S.D.).

**Figure 4.**
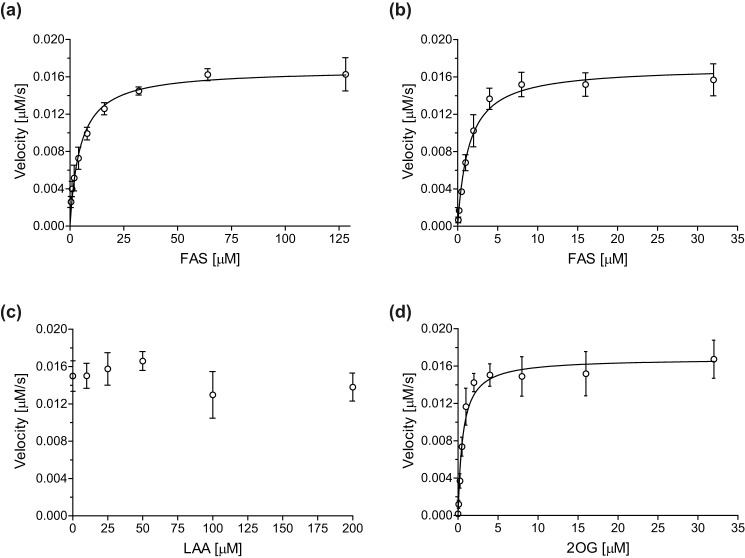
**Determination of steady-state kinetic parameters for AspH from initial hydroxylation rates of the thioether-linked cyclic peptide hFX–CP_101–119_ (Fig. 1*b*, *peptide 4*).** *a*, *K*_*m*_^app^ of AspH for FAS in the absence of LAA. *b*, *K*_*m*_^app^ of AspH for FAS in the presence of LAA. *c*, effect of LAA on AspH activity. *d*, *K*_*m*_^app^ of AspH for 2OG. The data are shown as the mean averages of three independent runs (*n* = 3; means ± S.D.). The results are summarized in Table 2. Initial peptide hydroxylation rates are shown in Fig. S6.

The apparent Michaelis constant (*K*_*m*_^app^) for the AspH co-factor Fe(II) was determined to be ∼4.8 μm using FAS as the iron source (Fig. 4*a* and Table 2). The *K*_*m*_^app^ of AspH for Fe(II) is in the range of those reported for bovine AspH (3 μm) (38) and other human asparaginyl and prolyl residue hydroxylases (30). This observation suggests that the unusual Fe(II)-binding site of AspH, which is composed of only two ligands (His_679_ and His_725_) rather than the typical three, is not reflected in an unusual *K*_*m*_^app^ value.

LAA is commonly added to assay buffers to enhance the activity of isolated 2OG oxygenases (*e.g.* for the procollagen and HIF-α prolyl hydroxylases) (46–48). In some cases LAA might act as a co-substrate, effectively replacing 2OG (*e.g.* certain TET (ten-eleven translocation) type oxygenases (55) and the plant enzyme ACCO (1-aminocyclopropane-1-carboxylate oxidase) (56, 57)). AspH activity was sensitive toward subtle changes in the redox environment, possibly because of redox active species formation in iron-containing buffers (58). LAA is a useful component of the AspH assay buffer because it improves assay robustness, possibly by scavenging reactive oxidizing species and/or maintaining the Fe(II) form of iron. The use of physiologically relevant concentrations of LAA (59) also increased assay accuracy when determining the *K*_*m*_^app^ for Fe(II) (Fig. 4*b*). In the absence of LAA, the *K*_*m*_^app^ for Fe(II) is approximately four times higher than in its presence (∼4.8 and ∼1.4 μm, respectively). However, when investigating the kinetic effect of LAA on AspH catalysis at saturating 2OG co-substrate and Fe(II) concentrations, LAA did not affect *k*_cat_ values within experimental error (Fig. 4*c* and Table 2).

Determining the *K*_*m*_^app^ for the AspH co-substrate 2OG by monitoring substrate hydroxylation is feasible as AspH-catalyzed 2OG turnover only proceeds at a low rate in the absence of substrate (36). The 2OG *K*_*m*_^app^ of AspH was determined to be ∼0.6 μm (Fig. 4*d* and Table 2). This is in the range of 2OG *K*_*m*_^app^ values reported for bovine AspH (∼5 μm) (38) and most other human 2OG oxygenases, including the PHDs and FIH (1–25 μm) (60). The 2OG *K*_*m*_^app^ of AspH is significantly smaller than reported cellular 2OG levels in healthy cells (61). It is also considerably less than the 2OG *K*_*m*_^app^ value of γ-butyrobetaine hydroxylase, the activity of which has the potential to be limited by *in vivo* 2OG availability (62).

**Figure 5.**
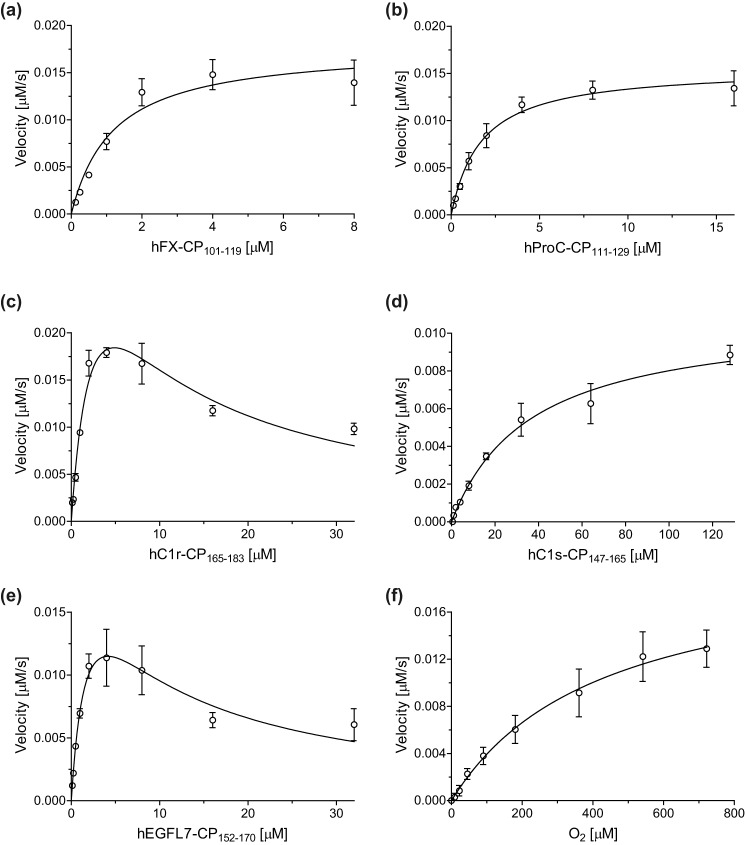
**Determination of steady-state kinetic parameters for AspH from initial hydroxylation rates of thioether-linked cyclic peptides.** *a*, *K_m_* of AspH for the cyclic peptide hFX–CP_101–119_ (Fig. 1*b*, *peptide 4*). *b*, *K_m_* of AspH for the cyclic peptide hProC–CP_111–129_. *c*, *K_m_* of AspH for the cyclic peptide hC1r–CP_165–183_. *d*, *K_m_* of AspH for the cyclic peptide hC1s–CP_147–165_. *e*, *K_m_* of AspH for the cyclic peptide hEGFL7–CP_152–170_. *f*, *K*_*m*_^app^ of AspH for O_2_ monitoring the hydroxylation of thioether-linked cyclic peptide hFX–CP_101–119_ (Fig. 1*b*, *peptide 4*). The data are shown as the mean averages of three independent runs (*n* = 3; means ± S.D.) and for *f* as the mean averages of four independent runs (*n* = 4; means ± S.D.). The results are summarized in Table 2. Initial peptide hydroxylation rates are shown in Figs. S6 and S7.

The *K_m_* of AspH for the thioether-linked cyclic peptide hFX–CP_101–119_ (Fig. 1*b*, *peptide 4*) was determined to be ∼1.2 μm (Fig. 5*a* and Table 2). This is lower than that reported for bovine AspH for an hFX EGFD1-derived peptide substrate (∼30 μm), which is likely a mixture of disulfide isomers (38). By contrast with previous observations using bovine AspH and an hFX EGFD1-derived peptide substrate (38), neither hFX–CP_101–119_ nor its hydroxylated product inhibited AspH activity at higher concentrations (up to 16 μm hFX–CP_101–119_). However, the values are difficult to compare because human hFX EGFD1 is not the natural substrate of bovine AspH. Further, variations in enzyme purity (partial for the bovine AspH *versus* high purified recombinant human AspH) and assay techniques (scintillation counting monitoring 2OG turnover (38) *versus* our SPE-MS) may result in different absolute values.

The combined results presented here and previously (36) reveal EGFD folding and/or disulfide isomerization influences AspH catalysis. The thioether-linked cyclic peptide hFX–CP_101–119_ (Fig. 1*b*, *peptide 4*) comprises the active site binding Cys 3–4 disulfide–containing element of EGFD AspH substrates, as well as the TPR domain–binding residues, as based on crystallographic analyses of AspH substrate complexes (Fig. S1), including with the noncanonical hFX EGFD1 disulfide isomer (Cys 1–2, 3–4, 5–6) (36). The cyclic structure of hFX–CP_101–119_ lacks the capability for disulfide isomerization that may complicate AspH hydroxylation kinetics. The cyclic thioether strategy thus seems to be a useful method for investigating the observed divergent hydroxylation levels of different AspH substrates that are observed in humans (Table 1).

Determining the *K_m_* and *k*_cat_ values for the different thioether-linked cyclic peptides enables rationalization of their observed relative hydroxylation efficiencies (Fig. 2). For example, both *K_m_* (∼1.7 μm) and *k*_cat_ (∼0.17 s^−1^) for hProC–CP_111–129_ are similar to those of hFX–CP_101–119_ (Fig. 5, *a* and *b*, and Table 2), suggesting similar hydroxylation efficiencies. These kinetic parameters are consistent with the similar EGFD hydroxylation levels of hFX and ProC that are observed in humans (Table 2) (6, 7, 49, 50, 63). In the case of the hC1r–CP_165–183_ and hEGFL7–CP_152–170_, substrate inhibition was observed at higher substrate concentrations (>4 μm; Fig. 5, *c* and *e*). This observation is in agreement with a previous report on substrate inhibition during the hydroxylation of hFX using bovine AspH (38). Nonetheless, the kinetic parameters suggest that similar *in vivo* EGFD Asp/Asn-hydroxylation levels might be anticipated for human EGFL7 and C1r. Human EGFD Asp/Asn-hydroxylation levels have been reported for C1r to be ∼90% (Table 1) (51, 52). Our kinetic parameters for hC1s–CP_147–165_ indicate that AspH has a considerably lower affinity for C1s (*K_m_* = ∼36.5 μm) and hydroxylates it slightly less efficiently (*k*_cat_ = ∼0.12 s^−1^) than hFX, ProC, and C1r, in accord with the reported *in vivo* results (51, 53). Kinetic parameters for the cyclic peptides hFVII–CP_121–139_ and hFIX–CP_108–126_ could not be determined because of their inefficient hydroxylation.

The experimental setup was modified to determine the *K*_*m*_^app^ for O_2_: the AspH *K*_*m*_^app^ for O_2_ was ∼426 μm under stationary condition using different partial pressures of O_2_ (Fig. 5*f* and Table 2). At higher partial pressures of O_2_, the standard deviation among independent quadruplicates increased slightly because of efficient peptide hydroxylation requiring short reaction times; however, maximum velocities (*v*_max_^app^) were still similar within experimental error, indicating good data quality and reproducibility (Fig. S7). The AspH *K*_*m*_^app^ for O_2_ is high compared with the values reported for most other human 2OG oxygenases and JmjC lysine demethylases (KDMs): only the *K*_*m*_^app^(O_2_) values of the HIF-α prolyl hydroxylases (PHD1–3), which range between 230 and 1746 μm (26–28), are considerably higher than the one of AspH. The *K*_*m*_^app^(O_2_) values for FIH (90 to 237 μm) (27, 30, 31), human phytanoyl-CoA hydroxylase (93 ± 43 μm) (27), the collagen prolyl 4-hydroxylases (40 μm) (26), KDM4A (57 ± 10 μm) (64), KDM4C (158 ± 13 μm) (64), KDM4E (197 ± 16 μm) (64), and KDM6A (180 ± 40 μm) (65) are all lower than for AspH and the PHDs. Under the identical experimental setup, the *K*_*m*_^app^(O_2_) values of FIH (∼110 μm) (32) and KDM4A (∼173 μm) (66) were at least two times lower than the AspH *K*_*m*_^app^ (Table 3), whereas PHD2 showed an apparently even weaker affinity for dioxygen (>450 μm) (32). Notably, although the *K*_*m*_^app^(O_2_) value for AspH appears relatively high, turnover number (*k*_cat_) comparison indicates a more efficient substrate hydroxylation for AspH compared with PHD2 under substrate saturating conditions (Table 3).

**Table 3:** **Reported Michaelis constants (*K*_*m*_^app^) and turnover numbers (*k*_cat_) of 2OG oxygenases for O_2_ using the same experimental setup as employed in our work**

	2OG oxygenase	Peptide substrate	*K*_*m*_^app^ for O_2_	*k*_cat_
			μ*m*	*s*^−*1*^
1	AspH	hFX–CP_101–119_	426 ± 73	0.23 ± 0.04
2	FIH (32)	HIF-1α CAD_35mer_*^a^*	110 ± 30	0.56 ± 0.04
3	PHD2 (32)	HIF-1α CODD*^b^*	460 ± 30	0.06 ± 0.01
4	PHD2 (32)	HIF-1α NODD*^c^*	>450	0.028 ± 0.001
5	KDM4A (66)	H3_1–15_K9me3	173 ± 23	ND*^d^*

*^a^* C-terminal transactivation domain (CAD, HIF-1α amino acids 789–822).

*^b^* C-terminal oxygen-dependent degradation domain (CODD, HIF-1α amino acids 556–574).

*^c^* N-terminal oxygen-dependent degradation domain (NODD, HIF-1α amino acids 395–413).

*^d^* ND, not determined.

### DICUSSION

Several lines of evidence suggest that AspH is a physiologically important 2OG oxygenase. These include its conserved nature in animals and likely presence in earlier organisms (36), links between mutations in the AspH encoding genes to inherited diseases (9–11), links of AspH to cancer (13, 15–17), mouse model studies (12), and analytical studies revealing extensive hydroxylation of (likely) AspH-catalyzed hydroxylation of EGFDs (3, 5–7, 67). Notably, the latter manifest to very different levels, suggesting the poised nature of AspH catalyzed hydroxylation (3, 6–8), a property that may be useful in a sensing or regulatory capacity. Work on the molecular roles of AspH has, however, been limited by a lack of robust assays for it in isolated form.

We developed a real-time AspH activity assay using SPE-MS and our recently reported soluble His_6_–AspH_315–758_ construct (36). The results with both disulfide fragments of hFX (Fig. 1*b*, *peptides 1/2* and *3*) and a thioether-linked cyclic peptide (hFX–CP_101–119_; Fig. 1*b*, *peptide 4*) support the proposal that AspH accepts EGFDs with the noncanonical (Cys 1–2, 3–4, 5–6) rather than the canonical (Cys 1–3, 2–4, 5–6) disulfide pattern. The thioether-linked cyclic peptide hFX–CP_101–119_ was identified as useful substrate to monitor AspH activity because its synthesis is straightforward, it is conformationally stable, and it is efficiently hydroxylated. Consistent with the prior literature (3), both Asp and Asn residues can be hydroxylated by AspH in an EGFD sequence context–dependent manner. Hydroxylation occurs at a single Asp/Asn residue, and hydroxylation can be readily quantified by SPE-MS analysis (Fig. 1 and Fig. S2).

The results support the proposal that SPE-MS is an excellent analytical technique for 2OG oxygenase assays: its high sensitivity requires only low enzyme and substrate concentrations for analysis, sample preparation is efficient because label-free MS-analysis is performed *in situ*, resulting in short overall measurement times (Fig. 1), and it does not suffer from the false positive/negative drawbacks of coupling 2OG oxygenase catalysis to other enzymes, using antibody-based detection or measuring co-substrate/co-product formation (68).

The SPE-MS assay was applied to determine kinetic parameters of human AspH for its substrates, Fe(II), and co-substrates (Figs. 4 and 5 and Table 2). There is the possibility that the kinetic parameters of AspH will vary depending on the substrate (sequence) identity, the form of the substrate, and/or the form of AspH. Indeed, there is precedent for each of these variables affecting 2OG oxygenase catalysis (69). For example, FIH, which like AspH accepts many substrates (and catalyzes the hydroxylation of both asparaginyl and aspartyl residues), manifests varying efficiencies with respect to its substrates both in isolated form and in cells (35, 70). The conformations of FIH substrates can also affect the efficiency of their hydroxylation (34). This conformational effect is even more strikingly evidenced in the preference of AspH for a noncanonical EGFD substrate disulfide pattern (Cys 1–2, 3–4, 5–6) rather than the canonical pattern (Cys 1–3, 2–4, 5–6), as observed in multiple crystal structures (36). Like AspH, where its TPR domain is important in catalysis (36), the activities of at least some JmjC KDMs are affected by noncatalytic domains, as exemplified by the cases of KDM7A/B (71). Thus, care should be taken in assuming that parameters determined for isolated truncated enzyme are necessarily relevant in a physiological context. Despite these caveats, the results of the initial kinetic characterization reported here on AspH are of interest, especially with respect to a potential role for AspH in redox regulation, including the hypoxic response.

The *K*_*m*_^app^ value of AspH for O_2_ is notably high compared with most other 2OG oxygenases for which data are reported (Table 3), with only the PHDs being more sensitive toward changes in O_2_ availability under substrate saturating conditions (26–28). Further, detailed kinetic studies on AspH are of interest including with respect to determining the molecular basis for its high *K*_*m*_^app^ for O_2_, which in the case of PHD2 (the most conserved and likely most important hypoxia sensor of the three human PHDs (72)) is proposed, on the basis of biophysical and kinetic studies, to result from slow binding of O_2_ to the active site Fe(II), potentially in part, because of a requirement to displace a tightly ligated water (29, 73). It should also be noted that the *k*_cat_(O_2_) value for AspH is substantially higher than that for PHD2 (Table 3). Nonetheless, coupled with the observation of varied levels of EGFD hydroxylation (3, 6–8), our observation of an unusually high *K*_*m*_^app^ of AspH with otherwise more typical kinetic parameters suggests that AspH could potentially play a role in redox regulation and potentially hypoxia sensing.

PHD2 binds Fe(II) and 2OG in an unusually stable manner (74), leading to the proposal the PHDs have evolved to focus on hypoxia sensing (although PHD activity can likely be limited by Fe(II) and, maybe, 2OG availability in some circumstances). Further work on AspH is required to determine the stability of its Fe(II)·2OG complex in the absence of substrate, although AspH does not catalyze 2OG oxidation efficiently in the absence of substrate (36). AspH is up-regulated in response to hypoxia at least in some cell lines (likely in a HIF-promoted manner) (75, 76, 77), potentially to compensate for reduction in AspH hydroxylase activity at lower O_2_ concentrations. Some other human 2OG oxygenases are up-regulated by hypoxia (77), including the hypoxia sensors PHD2 (78, 79) and, especially, PHD3 (75, 80). A potential role for AspH in hypoxic regulation is thus consistent with its observed strong up-regulation in hypoxia in cells (75, 76, 77), including in hypoxic tumors (13, 15, 81). Further investigations should be directed to determine AspH substrate hydroxylation levels *in vivo* as a function of O_2_ availability and disease. The results might help to decipher the mechanism of how AspH impacts on cell motility and the molecular function(s) of Asp/Asn hydroxylation.

In general, EGFDs manifest high sequence variability (82), which could explain differences in observed *in vivo* EGFD hydroxylation levels. This hypothesis is supported by the observation that, at least for the tested substrates, the AspH kinetic parameters for the stable thioether-linked cyclic peptides appear to reflect *in vivo* observed AspH substrate hydroxylation levels well (Tables 1 and 2). This observation means that thioether-linked cyclic peptides based on the sequences of AspH substrate EGFDs might be used to predict *in vivo* EGFD hydroxylation levels of AspH substrates that have not previously been validated *in vivo*. In this regard, the human EGFL7 protein, which is a target of HIF and is up-regulated in hypoxia (83) and which contains an EGFD bearing the AspH substrate consensus sequence, is one interesting example (84). EGFL7 is proposed to have roles in angiogenesis (85, 86) and the promotion of cell motility in human cancers (*e.g.* hepatocellular carcinoma (87), prostate cancer (88), and gastric cancer (89)). EGFL7 might thus constitute a disease-linked physiologically relevant AspH substrate (87–89). A cyclic peptide based on the sequence of EGFL7 is an efficient AspH substrate (Figs. 2 and 5*e*). Further cell-based experiments should be performed to investigate the biological relevance of this result.

EGFD disulfide isomerization may be of *in vivo* relevance with respect to AspH function (36). Indeed, the imperfect lack of correlation between the observed *in vitro* and *in vivo* AspH substrate hydroxylation levels may reflect complex regulatory factors, potentially involving context-dependent variations in disulfide patterns.

Several human 2OG oxygenases are being pursued as drug targets, with inhibitors of the PHDs being recently approved for the treatment of anemia in chronic kidney disease (90, 91). At least in part, the mode of action of the clinically used compound mildronate is proposed to involve inhibition of γ-butyrobetaine hydroxylase, thereby altering cellular metabolism (92). Although AspH is not yet a validated medicinal chemistry (anticancer) target, the efficient AspH activity assay together with the kinetic parameters of AspH reported here will be useful in designing an AspH inhibition assay to develop small molecule probes to investigate AspH function *in vivo*. The assay will also be useful in profiling clinically administered and clinical candidate 2OG oxygenase inhibitors, with a view to help enabling safe medicines. In this regard, it may be that the unusual active site chemistry of AspH can be exploited to obtain selectivity.

## Experimental procedures

### General information

All chemicals were obtained from commercial sources (Sigma–Aldrich) and used as received. Milli Q ultrapure (MQ-grade) water was used for buffers; LC-MS grade solvents were used for SPE-MS. Co-factor/co-substrate stock solutions (LAA: 50 mm in MQ-grade water; 2OG: 10 mm in MQ-grade water; ammonium iron(II) sulfate hexahydrate, FAS, (NH_4_)_2_Fe(SO_4_)_2_·6H_2_O: 400 mm in 20 mm HCl diluted to 1 mm in MQ-grade water) were freshly prepared from commercial solids each day AspH assays were performed.

### Recombinant AspH production and purification

Reported procedures were used (36). In brief, a pET-28a(+) vector encoding for N-terminal His_6_-tagged AspH_315–758_ (His_6_–AspH_315–758_) was transformed into *Escherichia coli* BL21 (DE3) cells. The resultant cells were grown in 2TY medium supplemented with kanamycin (0.05 mm) at 37 °C with shaking (180 rpm). AspH production was induced at an *A*_600_ of ∼1.2 at 18 °C by adding isopropyl β-d-thiogalactopyranoside (1 m) to a final concentration of 0.1 mm. The cells were shaken for 16 h at 18 °C and harvested by centrifugation (8000 rpm, 8 min, 4 °C); the resultant cell pellets were stored at −80 °C. Frozen cells were resuspended (30 g/100 ml) in ice-cold 50 mm HEPES buffer (pH 7.5, 500 mm NaCl, 5 mm imidazole) containing EDTA-free protease inhibitor mixture tablets (1 tablet/50 ml; Roche Diagnostics or Sigma–Aldrich) and DNase I (bovine pancreas, grade II; Roche Diagnostics). The cells were lysed by sonication on ice (eight 30-s bursts; Sonics Vibra-Cell VCX500, amplitude: 60%), and the lysates were then centrifuged (20,000 rpm, 30 min, 4 °C). The supernatant containing AspH was purified at 4 °C by Ni(II)-affinity chromatography (HisTrap HP column, GE Healthcare; 1 ml/min flow rate) using an ÄKTA Pure machine (GE Healthcare) with a gradient of wash (50 mm HEPES, pH 7.5, 500 mm NaCl, 40 mm imidazole) and elution buffers (50 mm HEPES, pH 7.5, 500 mm NaCl, 500 mm imidazole). Eluted fractions containing AspH were pooled, then concentrated using an Amicon Ultra centrifugal filter (4000 rpm, 4 °C), and further purified by size-exclusion chromatography using a HiLoad 26/60 Superdex 75 pg 300-ml column with a flow rate of 1 ml/min and 50 mm HEPES (pH 7.5, 150 mm NaCl) as elution buffer. AspH was >95% pure by SDS-PAGE analysis and had the anticipated mass as reported (36). His_6_–AspH_315–758_ was stored in 50 mm HEPES buffer (pH 7.5, 150 mm NaCl) at a concentration of 125 μm at −78 °C; fresh aliquots were used for all biochemical experiments.

### AspH substrates

AspH substrates were initially designed based on the sequence of EGFD1 of human coagulation factor X (hFX amino acids 86–124) (6, 7); all were prepared with a C-terminal amide. The hFX–EGFD1_86–124_ peptide (Fig. 1*b*, *peptide 1/2*) was synthesized by solid-phase peptide synthesis (SPPS) with the disulfide bridges being formed by thiol-oxidation in air-saturated buffer and purified by Peptide Synthetics (Peptide Protein Research Ltd, UK). The hFX–EGFD1_86–124_-4Ser peptide (Fig. 1*b*, *peptide 3*) was synthesized by SPPS and purified by GL Biochem (Shanghai) Ltd (Shanghai, China).

The thioether-linked cyclic peptide hFX–CP_101–119_ (hFX amino acids 101–119; Fig. 1*b*, *peptide 4*) was synthesized from the corresponding linear peptide (d-Ala replacing Cys101_hFX_ and Ser replacing Cys112_hFX_) which was obtained by SPPS using the Fmoc-protection strategy (36). Microwave-assisted SPPS was performed using an automated peptide synthesizer (Liberty Blue, CEM Corporation) from the C to N termini on Rink Amide MBHA resin (AGTC Bioproducts; loading: 0.6–0.8 mmol/g) using iterative coupling (90 °C; 140 s; *N*,*N*-diisopropylcarbodiimide, Oxyma, Hünig's base, Fmoc-protected amino acids) and deprotection steps (90 °C; 90 s; 80/20% (v/v) DMF/piperidine). The N-terminal amine of the linear peptide was capped on the resin using *N*-chloroacetylsuccinimide. The linear peptide was cleaved from the resin and deprotected using a mixture of TFA, triisopropylsilane, 1,3-dimethoxybenzene, and water (92.5/2.5/2.5/2.5% (v/v/v/v), respectively). The solids were separated, and the linear peptide was precipitated from the solution using cold diethyl ether. The solid linear peptide was dissolved in water/acetonitrile, lyophilized, dissolved in aqueous triethylammonium acetate buffer (1 m, pH 8.5)/acetonitrile, and cyclized in a microwave reactor (Biotage Initiator, 10 min at 80 °C) (93). The crude product was filtered and directly purified using a semipreparative HPLC machine (JASCO) equipped with a reverse-phase column (Gemini 00G-4454-U0-AX; phase NX-C18). A linear gradient (0–30% (v/v) over 35 min) of acetonitrile in deoxygenated MQ-grade water (each containing 0.1% (v/v) TFA) was used as eluent. Fractions containing the cyclic peptide hFX–CP_101–119_ (*t*_R_ = 34.4 min) were combined, lyophilized, and analyzed by MS.

The thioether-linked cyclic peptides hFVII–CP_121–139_, hFIX–CP_108–126_, hProC–CP_111–129_, hC1r–CP_165–183_, hC1s–CP_147–165_, hFXII–CP_110–128_, and hEGFL7–CP_152–170_ were designed based on the amino acid sequence of the EGFDs of the corresponding human proteins and prepared as described above. The details are given in Fig. S4.

### AspH activity assays

The substrate mixture (1.0 ml) containing 2.4 μm peptide, 120 μm LAA, 12 μm FAS, and 12 μm 2OG in 50 mm HEPES buffer (pH 7.5) was added into a well of a 2-ml volume 96-well assay plate (Greiner) at 20 °C under an ambient atmosphere. A blank sample was analyzed using a RapidFire RF 360 high-throughput sampling robot (Agilent) attached to an Agilent 6530 accurate mass quadrupole TOF mass spectrometer operated in the positive ionization mode. The enzyme mixture (0.2 ml), containing 0.6 μm His_6_–AspH_315–758_ in 50 mm HEPES buffer (pH 7.5), was then added to the well and thoroughly mixed. The RapidFire sampling robot was programmed to analyze 1 sample/min. The following SPE-MS conditions were used: assay samples were aspirated under vacuum for 0.4 s and loaded onto a C4 SPE cartridge. After loading, the C4 SPE cartridge was washed with 0.1% (v/v) aqueous formic acid to remove nonvolatile buffer salts (5 s, 1.5 ml/min). The peptide was then eluted from the SPE cartridge with 0.1% (v/v) aqueous formic acid in 85/15 (v/v) acetonitrile/water into the mass spectrometer (5 s, 1.25 ml/min). The SPE cartridge was re-equilibrated with 0.1% (v/v) aqueous formic acid (1 s, 1.25 ml/min). The mass spectrometer parameters were capillary voltage (3500 V), fragmentor voltage (150 V), gas temperature (350 °C), gas flow (12 liters/min). The *m*/*z* + 2 charge states of the substrate peptide and the product (hydroxylated) peptide were used to extract ion chromatogram data; peak areas were integrated using RapidFire Integrator software (Agilent). The data were exported into Microsoft Excel and used to calculate the percentage of conversion of the hydroxylation reaction using the equation: % conversion = 100 × (integral product peptide)/(integral substrate peptide + integral product peptide).

### Determination of kinetic parameters

Maximum velocities (*v*_max_ or *v*_max_^app^ monitoring hFX–CP_101–119_ turnover) and Michaelis constants (*K_m_* or *K*_*m*_^app^ monitoring hFX–CP_101–119_ turnover) of AspH were determined in independent triplicates for LAA, Fe(II), 2OG, and cyclic peptide AspH substrates by SPE-MS. An enzyme mixture (0.1 ml) containing 0.6 μm His_6_–AspH_315–758_ in 50 mm HEPES buffer (pH 7.5) was added at 20 °C to a substrate mixture (0.5 ml) containing peptide substrate and co-factors (1.2× final concentration) in 50 mm HEPES buffer (pH 7.5). Final substrate and co-factor/co-substrate concentrations are given in Fig. S6. The reactions were monitored with a rate of 1 sample/25 s using the same SPE-MS configuration as described above. The data were analyzed as described above, and the slopes of the initial reaction rates (Fig. S6) were fitted to a Michaelis–Menten plot using nonlinear regression (GraphPad Prism 5).

For determining *v*_max_^app^ and *K*_*m*_^app^ of AspH for O_2_, 2.2 μm hFX–CP_101–119_ (65 μl) in 50 mm HEPES (pH 7.5) were exposed in a gas-tight glass vial to variable O_2_ concentrations (in nitrogen; Fig. S7) using a mass flow controller (32). After equilibrating the atmosphere, co-factor and co-substrates were added by syringe (1.5 μl of 4.7 mm LAA and 0.99 mm 2OG in MQ-grade water and 1.5 μl of 0.99 mm FAS in MQ-grade water) followed by 2.0 μl of 3.5 μm His_6_–AspH_315–758_ in 50 mm HEPES (pH 7.5) at 20 °C. The enzyme reaction was stopped after the indicated reaction time by the addition of 15% (v/v) aqueous formic acid (4 μl) and analyzed by SPE-MS using the configurations given above; the experiments were performed in independent quadruplicates. The data were analyzed as described, and the slopes of the initial reaction rates (Fig. S7) were fitted to a Michaelis–Menten plot using nonlinear regression (GraphPad Prism 5).

To determine turnover numbers (*k*_cat_), the AspH active sites were titrated in independent triplicates using pyridine-2,4-dicarboxylic acid as a tight binding AspH inhibitor (38, 54). Final inhibitor concentrations (in DMSO) are given in Fig. S5. The enzyme mixture (0.1 ml), containing 0.6 μm His_6_–AspH_315–758_ in 50 mm HEPES buffer (pH 7.5), was incubated with 1% (v/v) DMSO inhibitor solution for 15 min at 20 °C. This was then added to the substrate mixture (0.5 ml), containing 2.4 μm hFX–CP_101–119_, 120 μm LAA, 2.4 μm FAS, 3.6 μm 2OG, and 1% (v/v) DMSO inhibitor solution in 50 mm HEPES buffer (pH 7.5). The reactions were monitored by SPE-MS with a rate of 1 sample/25 s using identical configurations as before. The data were analyzed as described, and the slopes of the initial reaction rates (Fig. S5) were fitted to a Morrison plot using nonlinear regression (GraphPad Prism 5) with the following constraints: 0 < enzyme active sites ([E]_T_) < 0.1 μm; *K*_*m*_^app^ (2OG) = 0.6 μm; concentration (2OG) = 3.0 μm. The Morrison equation is as follows, where *I* is the inhibitor concentration, *K_i_* is the dissociation constant of the inhibitor, E_T_ is the total concentration of active enzyme, and *v*_i_/*v*_0_ is the fractional enzyme activity (94).

(Eq. 1) $$\frac{v_{i}}{v_{0}}=\left(1-\frac{{\left[\text{E}\right]}_{\text{T}}+\left[I\right]+(K_{i}\cdot \left(1+\left[\text{S}\right]/K_{m,\text{app}}\right)-\sqrt{{\left({\left[\text{E}\right]}_{\text{T}}+\left[I\right]+K_{i}\cdot \left(1+\left[\text{S}\right]/K_{m,\text{app}}\right)\right)}^{2}-4{\left[\text{E}\right]}_{\text{T}}\left[\text{I}\right]}}{2{\left[\text{E}\right]}_{\text{T}}}\right)$$

## Supplementary Material

Supporting Information

## Footnotes

2 The abbreviations used are:

2OG: 2-oxoglutarate
AspH: aspartate/asparagine–β-hydroxylase
EGFD: epidermal growth factor–like domain
EGFL7: epidermal growth factor–like protein 7
FAS: ferrous ammonium sulfate
FIH: factor-inhibiting HIF
hFX: human coagulation factor X
HIF: hypoxia-inducible transcription factor
LAA: l-ascorbic acid
SPE: solid-phase extraction
PHD: HIF prolyl hydroxylase
TPR: tetratricopeptide repeat
KDM: lysine demethylase
SPPS: solid-phase peptide synthesis
Fmoc: *N*-(9-fluorenyl)methoxycarbonyl.
