# Supplementary material for: Kinetic parameters of human aspartate/asparagine–β-hydroxylase suggest that it has a possible function in oxygen sensing
Source: J Biol Chem. 2020 Feb 26;295(23):7826–38. doi: 10.1074/jbc.RA119.012202 (PMC7278358; doi:10.1074/jbc.RA119.012202)
Supplement: Supporting Information [file supp_295_23_7826__index.html]

Kinetic parameters of human aspartate/asparagine-β-hydroxylase suggest that it has a possible function in oxygen sensing — Kinetic parameters of aspartate/asparagine-β-hydroxylase — Kinetic parameters of human aspartate/asparagine–β-hydroxylase suggest that it has a possible function in oxygen sensing — Kinetic parameters of aspartate/asparagine–β-hydroxylase — Supporting Information 

# Kinetic parameters of human aspartate/asparagine–β-hydroxylase suggest that it has a possible function in oxygen sensing

## Supporting Information

- Supporting Information (to be published online) - Supporting Information
