# Supplementary material for: Kinetic parameters of human aspartate/asparagine–β-hydroxylase suggest that it has a possible function in oxygen sensing
Source: J Biol Chem. 2020 Feb 26;295(23):7826–38. doi: 10.1074/jbc.RA119.012202 (PMC7278358; doi:10.1074/jbc.RA119.012202)
Supplement: Supporting Information [file supp_RA119.012202_157558_2_supp_480527_q67thh.pdf]

Supporting Information

Kinetic parameters of human aspartate/asparagine-β-hydroxylase suggest that it has a possible function in oxygen sensing

Lennart Brewitz, Anthony Tumber, and Christopher J. Schofield*

Chemistry Research Laboratory, University of Oxford, 12 Mansfield Road, OX1 3TA, Oxford, United Kingdom.

Email: christopher.schofield@chem.ox.ac.uk

Figure S1. Views from a crystal structure of AspH:hFX-CP$_{101-119}$ (PDB entry: 6RK9). Color code: grey: His$_6$-AspH$_{315-758}$; yellow: carbon-backbone of *N*-oxalylglycine (NOG); green: carbon-backbone of the hFX-CP$_{101-119}$ peptide; violet: Mn; red: oxygen; blue: nitrogen; pale yellow: sulfur. w: water.

(a) The AspH:hFX-CP$_{101-119}$ crystal structure (PDB entry: 6RK9) contains two His$_6$-AspH$_{315-758}$ molecules in the asymmetric unit (1). An overview of the AspH protein fold complexed to the hFX-CP$_{101-119}$ peptide is shown. Fe(II) is substituted by Mn, and 2-oxoglutarate by NOG; (b) Close-up of the AspH active site: key residues engaged in substrate and co-substrate binding are shown. Two alternative conformations of Asp103$_{hFX}$ are present in the crystal structure (productive conformation in green, distance Cβ(Asp103$_{hFX}$)-Mn: 4.3 Å; non-productive conformation in olive); (c) Close-up of the AspH hydrophobic pocket composed of the hydrophobic tetratricopeptide repeat domain (TPR) residues Val462, Leu465, Leu466, Phe496, Ile497, and Phe527 (highlighted in light pink) which buries the hFX-CP$_{101-119}$ peptide Tyr108$_{hFX}$ residue. His493 and Arg526 of the AspH-TPR domain are positioned to interact with Tyr108$_{hFX}$ (3.0 and 2.8 Å, respectively); (d) Surface representation (grey) of the AspH:hFX-CP$_{101-119}$ crystal structure showing the hydrophobic TPR pocket to which the essential AspH substrate consensus sequence residue Tyr108$_{hFX}$ binds (His$_6$-AspH$_{315-758}$ in magenta).

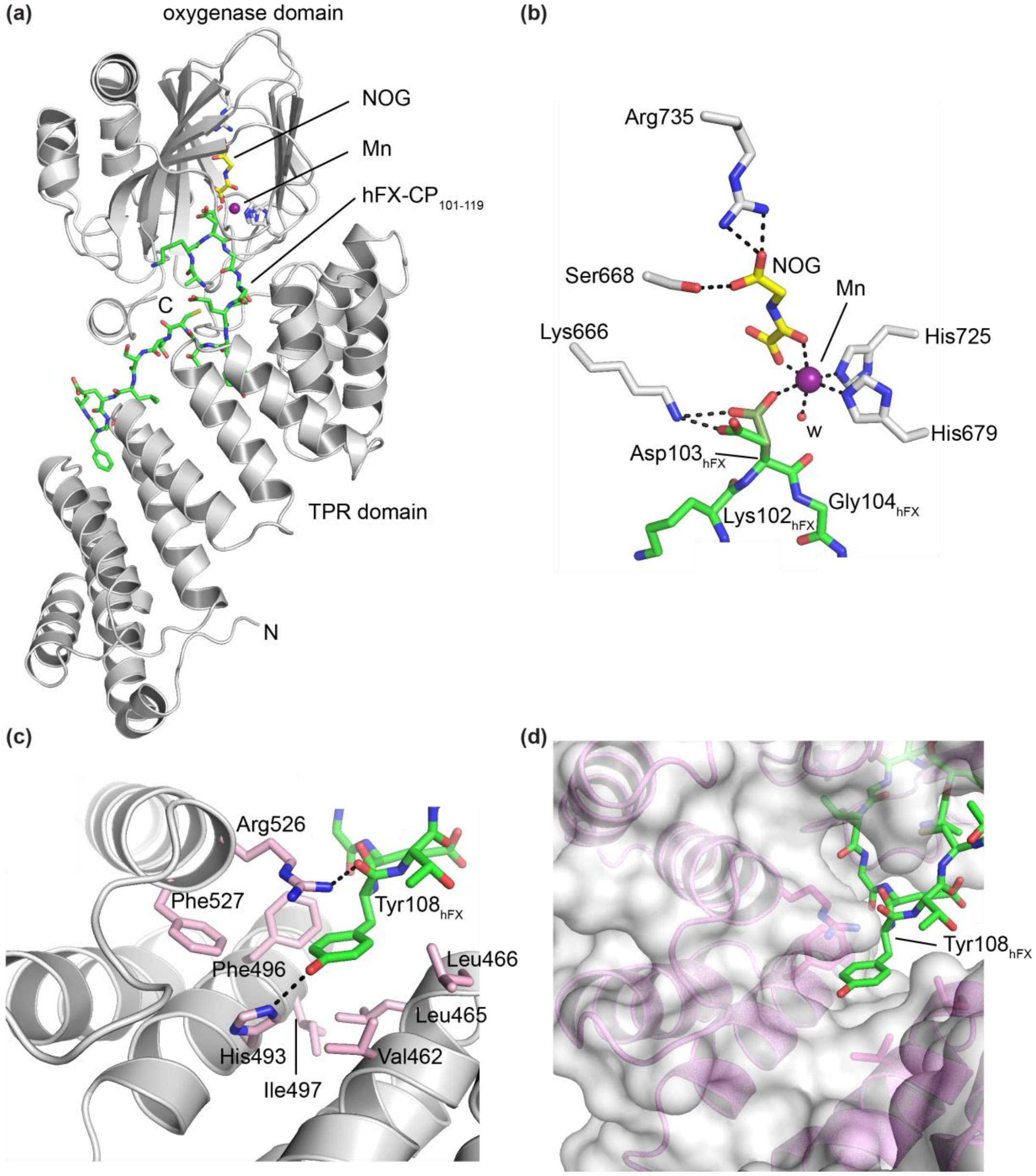

Figure S2. Optimization of AspH assay conditions. Assays were performed as described in the Experimental Procedures in the presence of His$_6$-AspH$_{315-758}$, hFX-CP$_{101-119}$ (4, Figure 1b), 100 μM L-ascorbic acid (LAA), 10 μM ferrous ammonium sulphate (FAS), and 10 μM 2-oxoglutarate (2OG), using solid-phase extraction coupled to mass spectrometry (SPE-MS) analysis at 20 °C. Each time point represents the % conversion calculated using the equation: % conversion = 100 x [integral hFX-CP$_{101-119}$(O) (product)] / [integral hFX-CP$_{101-119}$ (substrate) + integral hFX-CP$_{101-119}$(O) (product)]. (a) Optimization of the His$_6$-AspH$_{315-758}$ concentration and enzyme-substrate (E/S) ratio in 50 mM Tris buffer (pH 7.5, 100 mM NaCl); (b) Effects of pH and NaCl on AspH catalysis in 50 mM MES buffer using 0.2 μM His$_6$-AspH$_{315-758}$ and 2.0 μM hFX-CP$_{101-119}$; (c) Effects of pH and NaCl on AspH catalysis in 50 mM Tris buffer using 0.2 μM His$_6$-AspH$_{315-758}$ and 2.0 μM hFX-CP$_{101-119}$; (d) Effects of pH and NaCl on AspH catalysis in 50 mM HEPES buffer using 0.2 μM His$_6$-AspH$_{315-758}$ and 2.0 μM hFX-CP$_{101-119}$. The final optimized assay conditions were: 0.1 μM His$_6$-AspH$_{315-758}$ and 2.0 μM hFX-CP$_{101-119}$ in 50 mM HEPES buffer (pH 7.5, 20 °C).

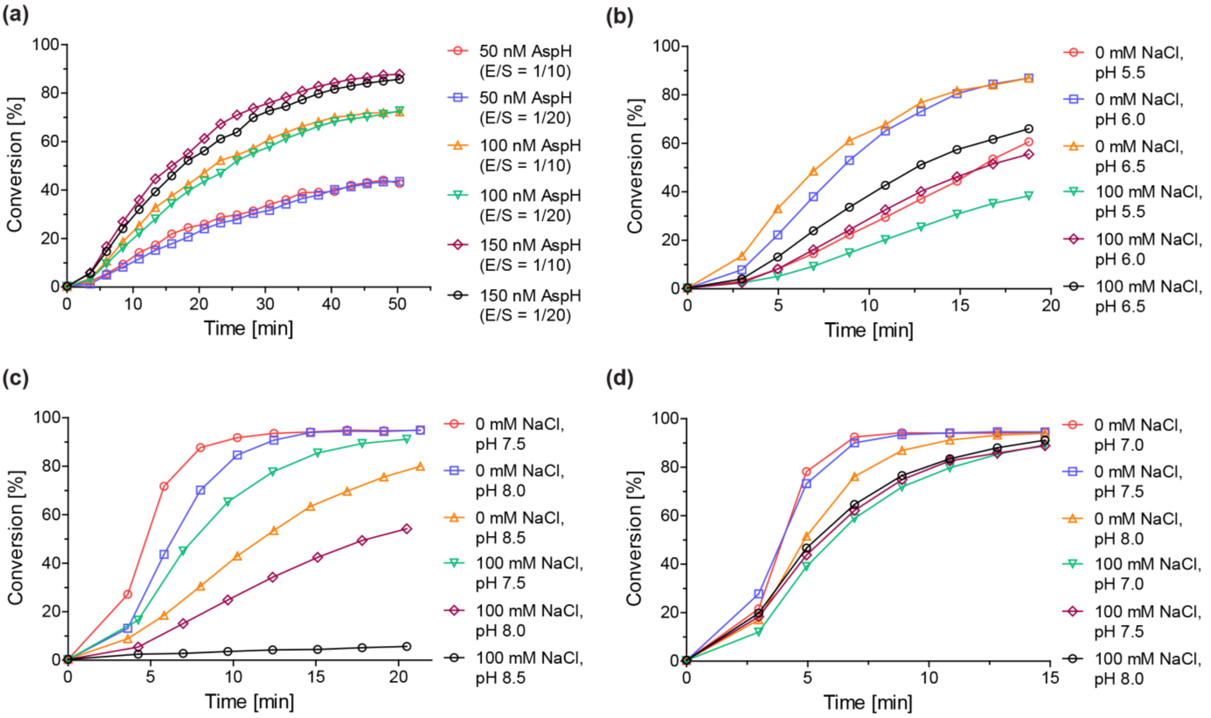

Figure S3. Quantification of AspH-catalyzed peptide hydroxylation using solid-phase extraction coupled to MS. Assay were performed as described in the Experimental Procedures using 0.1 µM His_6-$AspH_{315\text{-}758}$, 2.0 µM hFX-$CP_{101\text{-}119}$ (4, Figure 1b), 100 µM LAA, 10 µM FAS, and 10 µM 2OG in 50 mM HEPES (pH 7.5, 20 °C). The AspH-hydroxylation site is in red, the cysteine sulfur in green, and substituted residues in light blue (D-Ala replacing $Cys101_{hFX}$ and Ser replacing $Cys112_{hFX}$). Numbering is according to the sequence of the AspH substrate EGFD1 of the human coagulation factor X (hFX). (a) Mass spectrum of the reaction mixture before the addition of His_6-$AspH_{315\text{-}758}$ (t = 0 min). Both HEPES (239.1 Da, m/z +1) and the cyclic peptide hFX-$CP_{101\text{-}119}$ (1022.5 Da, m/z +2; 682.3 Da, m/z +3) were observed; (b) Mass spectrum of the reaction mixture after the addition of His_6-$AspH_{315\text{-}758}$; the reaction was complete 7 min after AspH addition as indicated by solid-phase extraction coupled to MS (t = 7 min). Both HEPES (239.1 Da, m/z +1) and the hydroxylated cyclic peptide hFX-$CP_{101\text{-}119}$(O) (1030.5 Da, m/z +2; 687.7 Da, m/z +3) were observed; there was no evidence for other oxidation products; (c) Time course of the AspH-catalyzed hydroxylation reaction. Each point represents the conversion calculated using the equation: % conversion = 100 x [integral product] / [integral substrate + integral product]; (d) The sum of the ion counts of substrate (green circles) and product (red squares) peptides is near constant throughout the reaction time course (black triangles), indicating that peptide ion counts can be used to quantify reaction progress. Note: The presence of AspH suppresses ionization of hFX-$CP_{101\text{-}119}$.

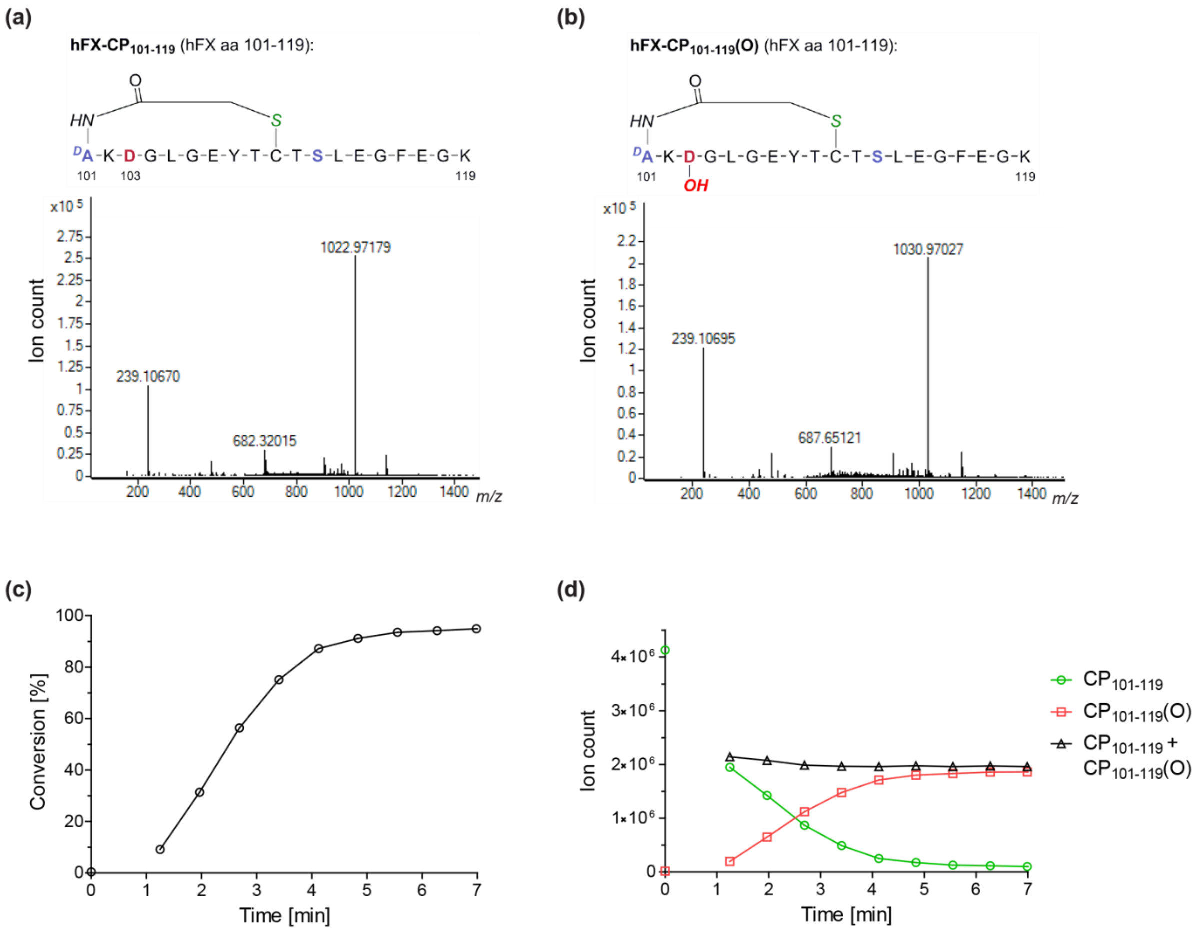

Figure S4. Synthesis and AspH-catalyzed hydroxylation of the thioether linked cyclic peptides (continues on the following 7 pages). The thioether linked cyclic peptides were synthesized and purified according to the exemplary description in the Experimental Procedures for the cyclic peptide hFX-$CP_{101-119}$. Assays were performed as described in the Experimental Procedures using 0.1 μM His_6-$AspH_{315-758}$, 2.0 μM cyclic peptide, 100 μM LAA, 10 μM FAS, and 10 μM 2OG in 50 mM HEPES (pH 7.5, 20 °C). The AspH hydroxylation sites are in red, the cysteine sulfur in green, and substituted residues in light blue (D-Ala replacing Cys-3 and Ser replacing Cys-5). Numbering is according to the sequence of the AspH-substrate EGFD of the wild-type human protein. Measurement times were normalized to the first sample injection analyzed after the addition of the Enzyme Mixture to the Substrate Mixture (t = 0 min), by which time low levels of hydroxylation were manifest.

(a) The thioether linked cyclic peptide hFX-$CP_{101-119}$ is based on the EGFD1 amino acid sequence of human coagulation factor X. 1) Sequence, structure, and analytic characteristics of hFX-$CP_{101-119}$. 2) Mass spectrum (SPE-MS) of the reaction mixture containing hFX-$CP_{101-119}$ before the addition of His_6-$AspH_{315-758}$ (t = 0 min). Both HEPES (239.1 Da, m/z +1) and hFX-$CP_{101-119}$ (1022.5 Da, m/z +2; 682.3 Da, m/z +3) were observed; the enlarged region shows the major m/z +2 peak. 3) Mass spectrum (SPE-MS) of the same reaction mixture 15 min after the addition of His_6-$AspH_{315-758}$; the conversion was >95%. Both HEPES (239.1 Da, m/z +1) and the hydroxylated cyclic peptide hFX-$CP_{101-119}$(O) (1030.5 Da, m/z +2; 687.7 Da, m/z +3) were observed; the enlarged region shows the major m/z +2 peak.

(1) **hFX-$CP_{101-119}$** (hFX amino acids 101-119):

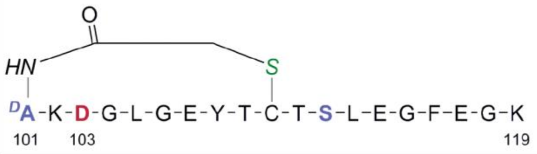

HPLC gradient: 0% to $30\%_{v/v}$ MeCN in H_2O over 35 min (t_R: 34.4 min)

HRMS (ESI): m/z calculated for $C_{88}H_{135}N_{22}O_{32}S$ $[M+H]^+$: 2043.9328, found: 2043.9347

(2) t = 0 min

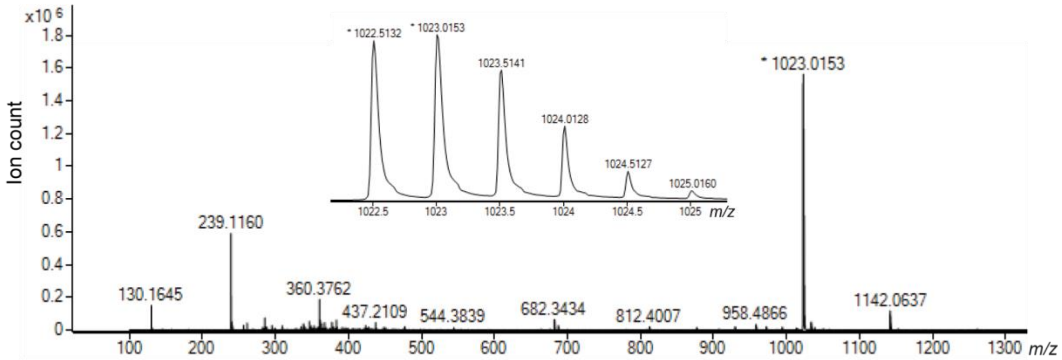

(3) t = 15 min

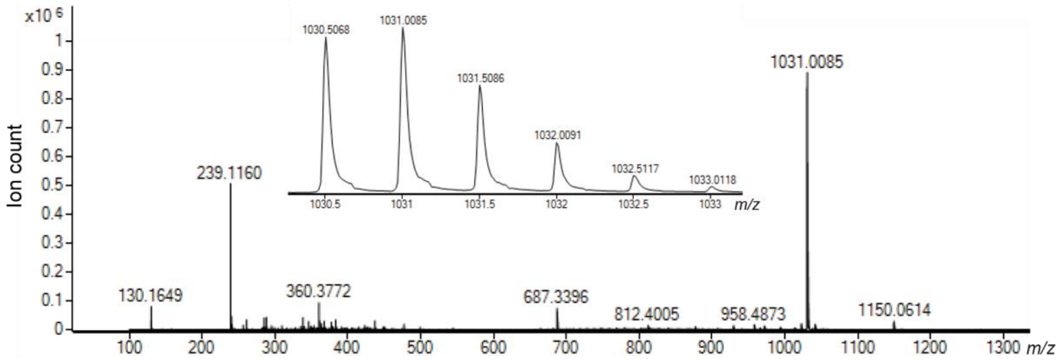

(b) The thioether linked cyclic peptide hFVII-CP$_{121\text{-}139}$ is based on the EGFD1 amino acid sequence of human coagulation factor VII. 1) Sequence, structure, and analytic characteristics of hFVII-CP$_{121\text{-}139}$. 2) Mass spectrum (SPE-MS) of the reaction mixture containing hFVII-CP$_{121\text{-}139}$ before the addition of His$_6$-AspH$_{315\text{-}758}$ (t = 0 min). Both HEPES (239.1 Da, m/z +1) and hFVII-CP$_{121\text{-}139}$ (1106.6 Da, m/z +2; 738.1 Da, m/z +3) were observed; the enlarged region shows the major m/z +2 peak. 3) Mass spectrum (SPE-MS) of the same reaction mixture 200 min after the addition of His$_6$-AspH$_{315\text{-}758}$; the conversion was ~40%. HEPES (239.1 Da, m/z +1), hFVII-CP$_{121\text{-}139}$ (1106.6 Da, m/z +2; 738.1 Da, m/z +3), and the hydroxylated cyclic peptide hFVII-CP$_{121\text{-}139}$ (O) (1114.6 Da, m/z +2; 743.4 Da, m/z +3) were observed; the enlarged region shows the major m/z +2 peaks.

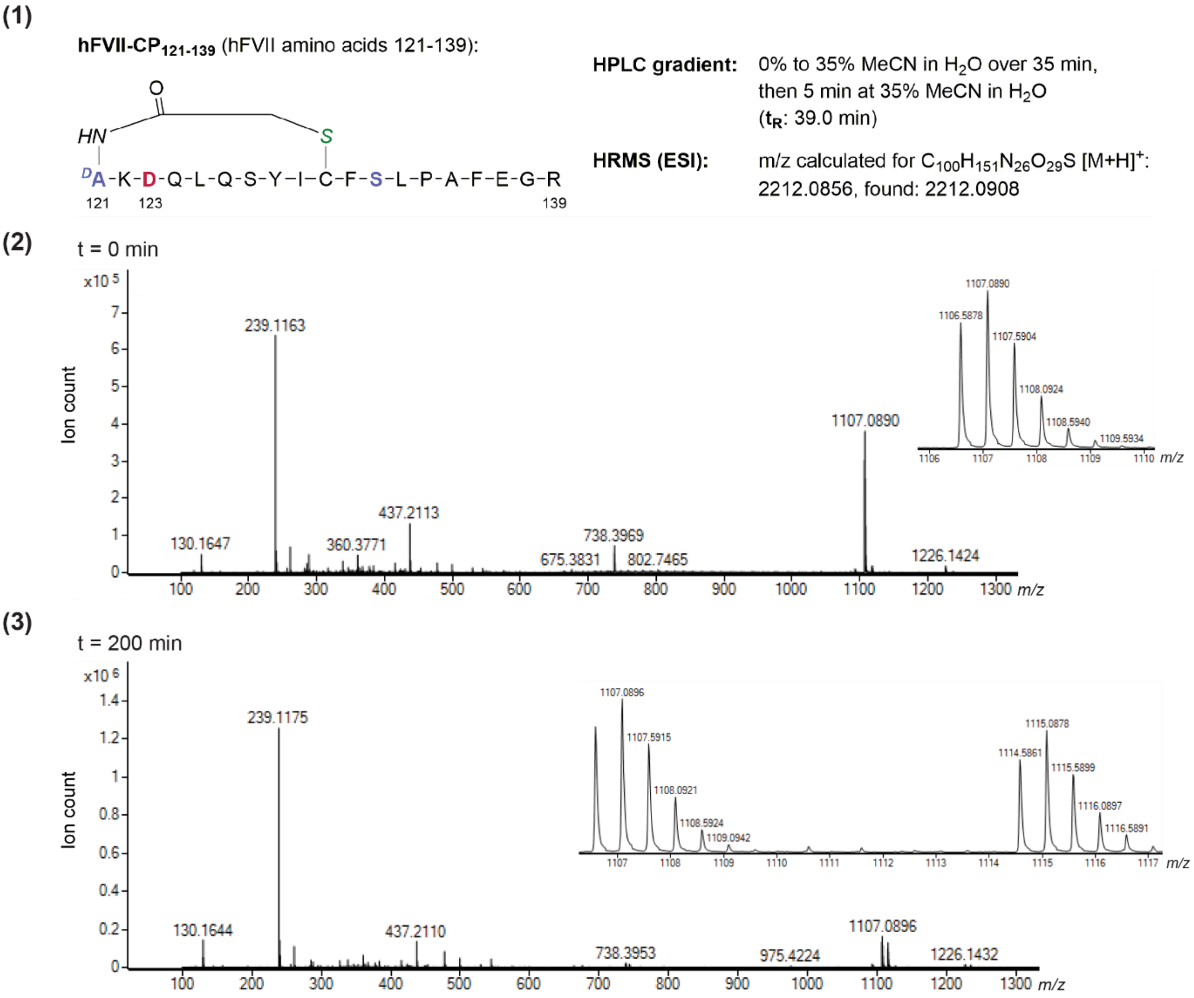

(c) The thioether linked cyclic peptide hFIX-CP$_{108\text{-}126}$ is based on the EGFD1 amino acid sequence of human coagulation factor IX. 1) Sequence, structure, and analytic characteristics of hFIX-CP$_{108\text{-}126}$. 2) Mass spectrum (SPE-MS) of the reaction mixture containing hFIX-CP$_{108\text{-}126}$ before the addition of His$_6$-AspH$_{315\text{-}758}$ (t = 0 min). Both HEPES (239.1 Da, m/z +1) and hFIX-CP$_{108\text{-}126}$ (1116.5 Da, m/z +2) were observed; the enlarged region shows the m/z +2 peak. 3) Mass spectrum (SPE-MS) of the same reaction mixture 200 min after the addition of His$_6$-AspH$_{315\text{-}758}$; the conversion was ~60%. HEPES (239.1 Da, m/z +1), hFIX-CP$_{108\text{-}126}$ (1116.5 Da, m/z +2), and the hydroxylated cyclic peptide hFIX-CP$_{108\text{-}126}$(O) (1124.5 Da, m/z +2) were observed; the enlarged region shows the m/z +2 peaks.

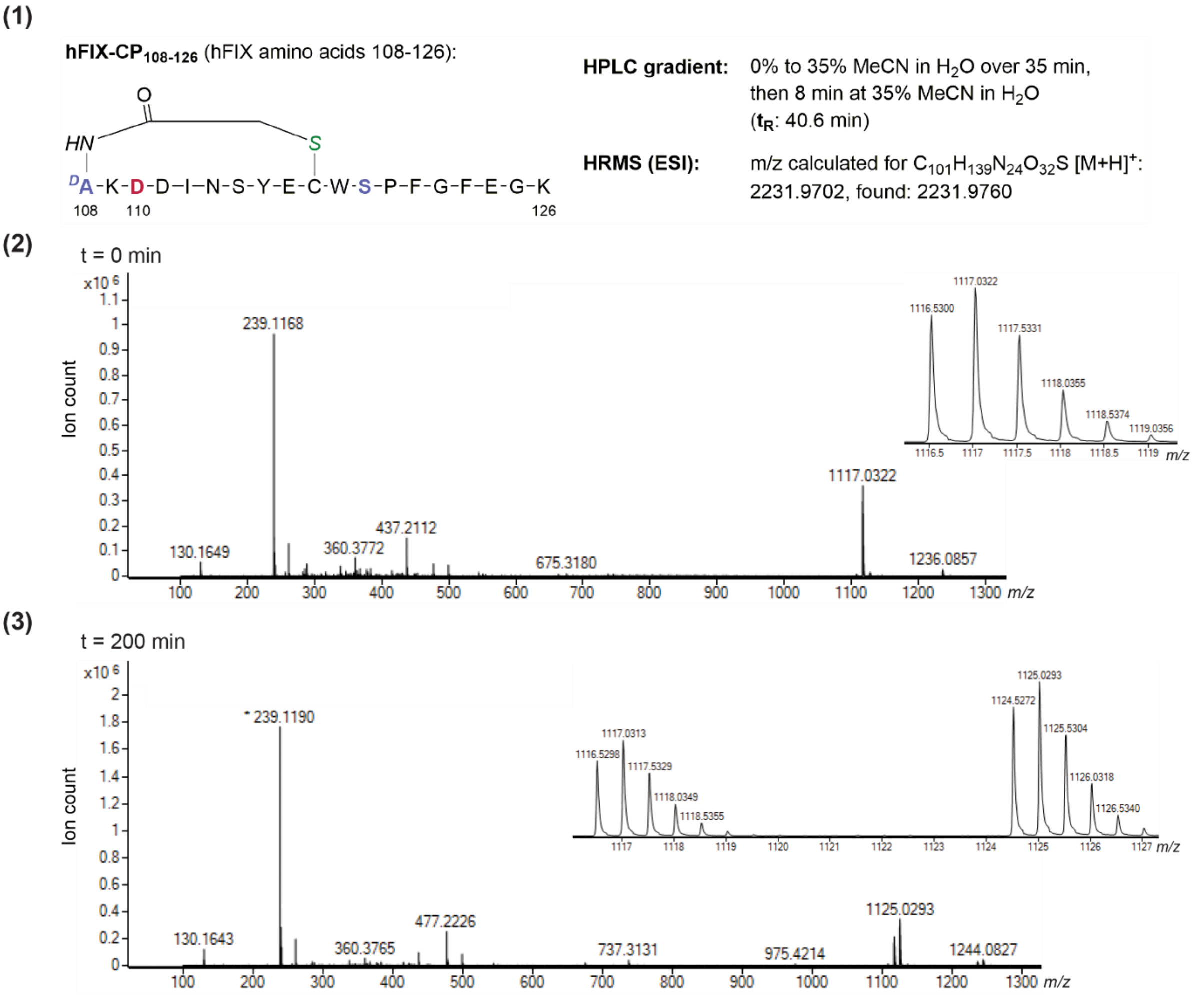

(d) The thioether linked cyclic peptide hFXII-CP$_{110\text{-}128}$ is based on the EGFD1 amino acid sequence of human coagulation factor XII. 1) Sequence, structure, and analytic characteristics of hFXII-CP$_{110\text{-}128}$. 2) Mass spectrum (SPE-MS) of the reaction mixture containing hFXII-CP$_{110\text{-}128}$ before the addition of His$_6$-AspH$_{315\text{-}758}$ (t = 0 min). Both HEPES (239.1 Da, m/z +1) and hFXII-CP$_{110\text{-}128}$ (1000.0 Da, m/z +2; 667.0 Da, m/z +3) were observed; the enlarged region shows the major m/z +2 peak. 3) Mass spectrum (SPE-MS) of the same reaction mixture 200 min after the addition of His$_6$-AspH$_{315\text{-}758}$; no oxidation of Asn112$_{\text{hFXII}}$ was observed; the enlarged region shows the m/z +2 peak.

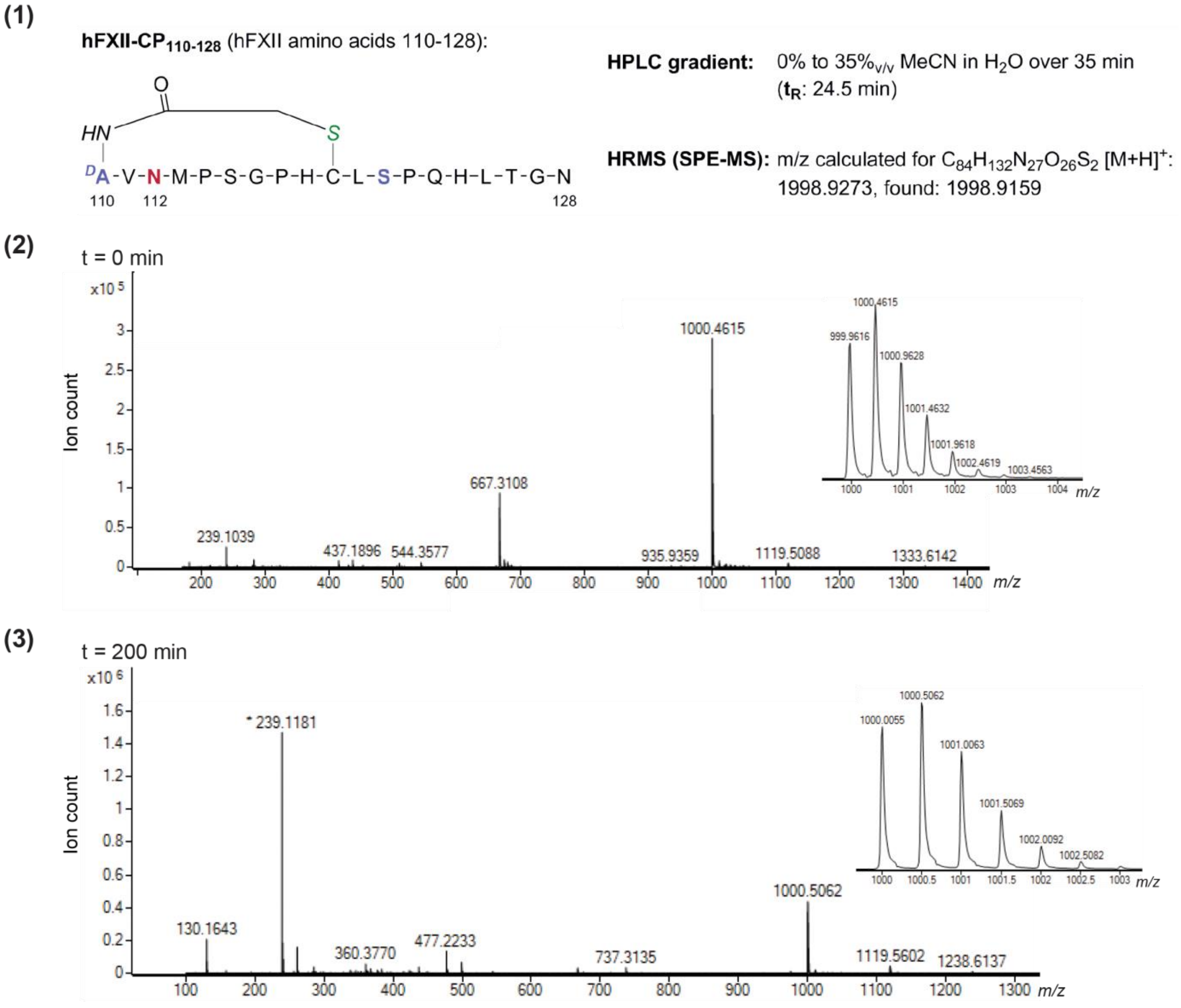

(e) The thioether linked cyclic peptide hProC-CP$_{111\text{-}129}$ is based on the EGFD1 amino acid sequence of human protein C. 1) Sequence, structure, and analytic characteristics of hProC-CP$_{111\text{-}129}$. 2) Mass spectrum (SPE-MS) of the reaction mixture containing hProC-CP$_{111\text{-}129}$ before the addition of His$_6$-AspH$_{315\text{-}758}$ (t = 0 min). Both HEPES (239.1 Da, m/z +1) and hProC-CP$_{111\text{-}129}$ (1020.0 Da, m/z +2) were observed; the enlarged region shows the m/z +2 peak. 3) Mass spectrum (SPE-MS) of the same reaction mixture 15 min after the addition of His$_6$-AspH$_{315\text{-}758}$; the conversion was >95%. Both HEPES (239.1 Da, m/z +1) and the hydroxylated cyclic peptide hProC-CP$_{111\text{-}129}$ (O) (1028.0 Da, m/z +2) were observed; the enlarged region shows the m/z +2 peak.

(1)

hProC-CP$_{111\text{-}129}$ (hProC amino acids 111-129):

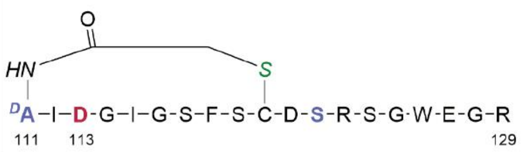

HPLC gradient: 0% to 30% MeCN in H_2O over 35 min, then 5 min at 30% MeCN in H_2O (**t$_R$**: 37.6 min)

HRMS (ESI): m/z calculated for $C_{85}H_{128}N_{27}O_{30}S$ $[M+H]^+$: 2038.9036, found: 2038.9092

(2)

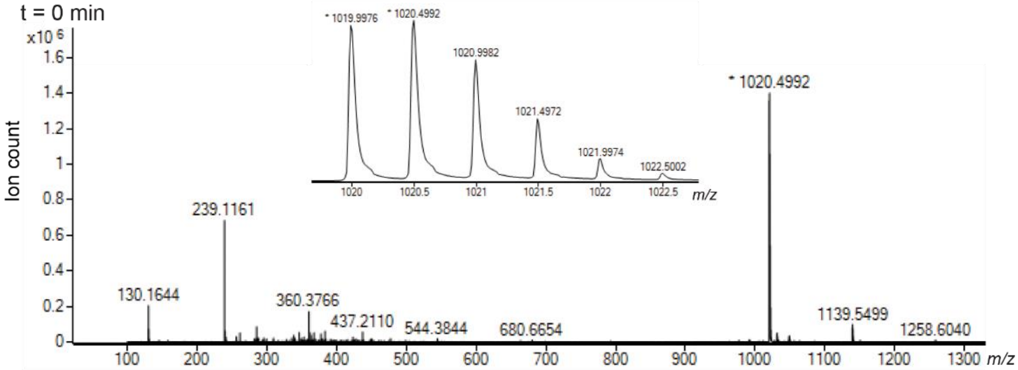

(3)

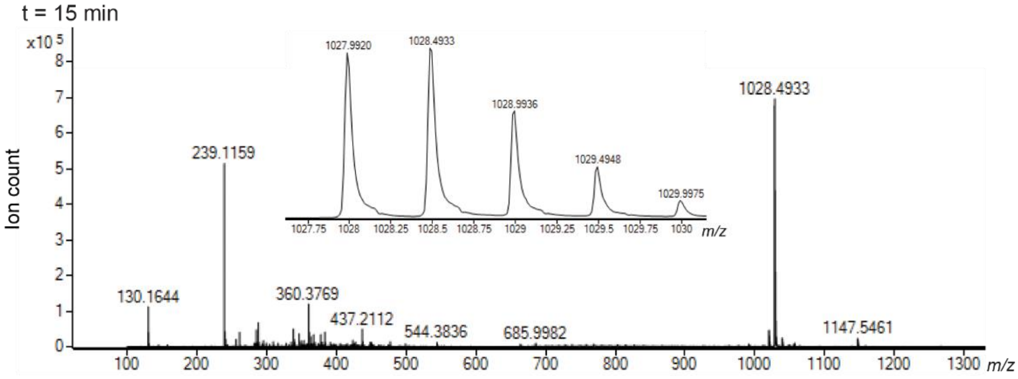

(f) The thioether linked cyclic peptide hC1r-CP$_{165\text{-}183}$ is based on the EGFD amino acid sequence of human complement C1r subcomponent. 1) Sequence, structure, and analytic characteristics of hC1r-CP$_{165\text{-}183}$. 2) Mass spectrum (SPE-MS) of the reaction mixture containing hC1r-CP$_{165\text{-}183}$ before the addition of His$_6$-AspH$_{315\text{-}758}$ (t = 0 min). Both HEPES (239.1 Da, m/z +1) and hC1r-CP$_{165\text{-}183}$ (1094.0 Da, m/z +2; 729.7 Da, m/z +3) were observed; the enlarged region shows the major m/z +2 peak. 3) Mass spectrum (SPE-MS) of the same reaction mixture 15 min after the addition of His$_6$-AspH$_{315\text{-}758}$; the conversion was ~90%. HEPES (239.1 Da, m/z +1), hC1r-CP$_{165\text{-}183}$ (1094.0 Da, m/z +2; 729.3 Da, m/z +3), and the hydroxylated cyclic peptide hC1r-CP$_{165\text{-}183}$ (O) (1102.0 Da, m/z +2; 735.0 Da, m/z +3) were observed; the enlarged region shows the major m/z +2 peaks.

(1)

hC1r-CP$_{165\text{-}183}$ (hC1r amino acids 165-183):

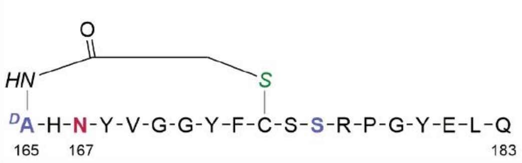

HPLC gradient: 0% to 35% MeCN in H_2O over 35 min ($\mathbf{t_R}$: 31.9 min)

HRMS (ESI): m/z calculated for $C_{98}H_{136}N_{27}O_{29}S$ $[M+H]^+$: 2186.9712, found: 2186.9766

(2)

(3)

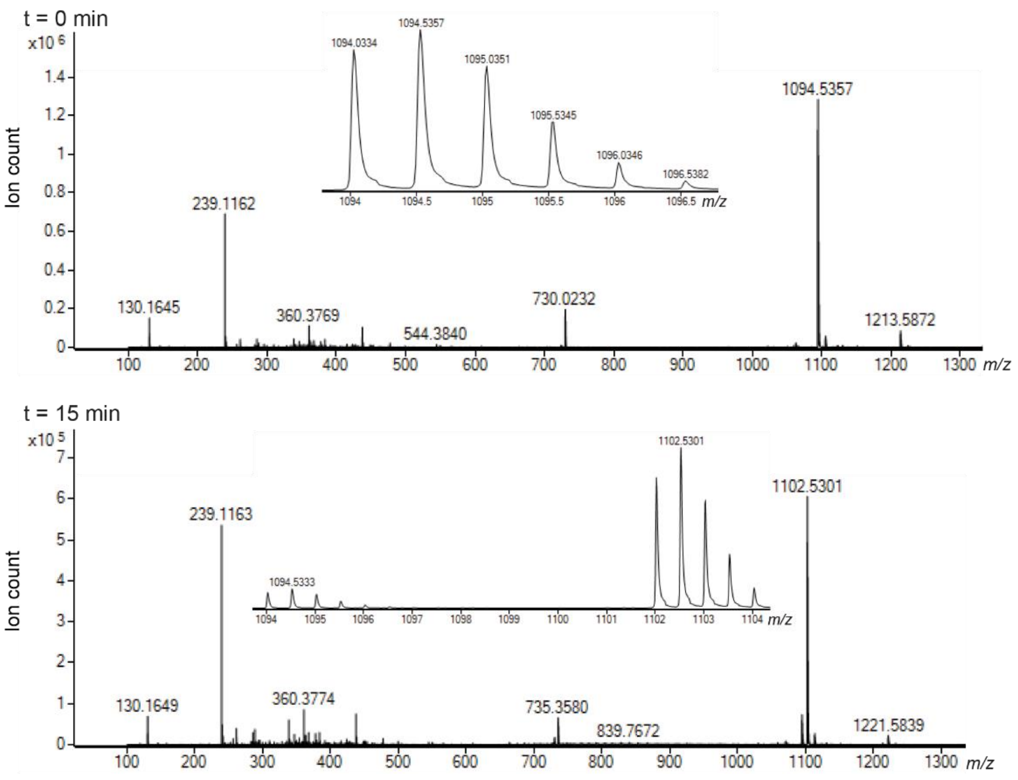

(g) The thioether linked cyclic peptide hC1s-$CP_{147\text{-}165}$ is based on the EGFD amino acid sequence of human complement C1s subcomponent. 1) Sequence, structure, and analytic characteristics of hC1s-$CP_{147\text{-}165}$. 2) Mass spectrum (SPE-MS) of the reaction mixture containing hC1s-$CP_{147\text{-}165}$ before the addition of His_6-$AspH_{315\text{-}758}$ (t = 0 min). Both HEPES (239.1 Da, m/z +1) and hC1s-$CP_{147\text{-}165}$ (1101.5, 1112.5 Da, m/z +2) were observed; the enlarged region shows the m/z +2 peak. 3) Mass spectrum (SPE-MS) of the same reaction mixture 200 min after the addition of His_6-$AspH_{315\text{-}758}$; the conversion was ~60%. HEPES (239.1 Da, m/z +1), hC1s-$CP_{147\text{-}165}$ (1101.5 Da, m/z +2), and the hydroxylated cyclic peptide hC1s-$CP_{147\text{-}165}$(O) (1109.5, 1120.5 Da, m/z +2) were observed; the enlarged region shows the m/z +2 peaks.

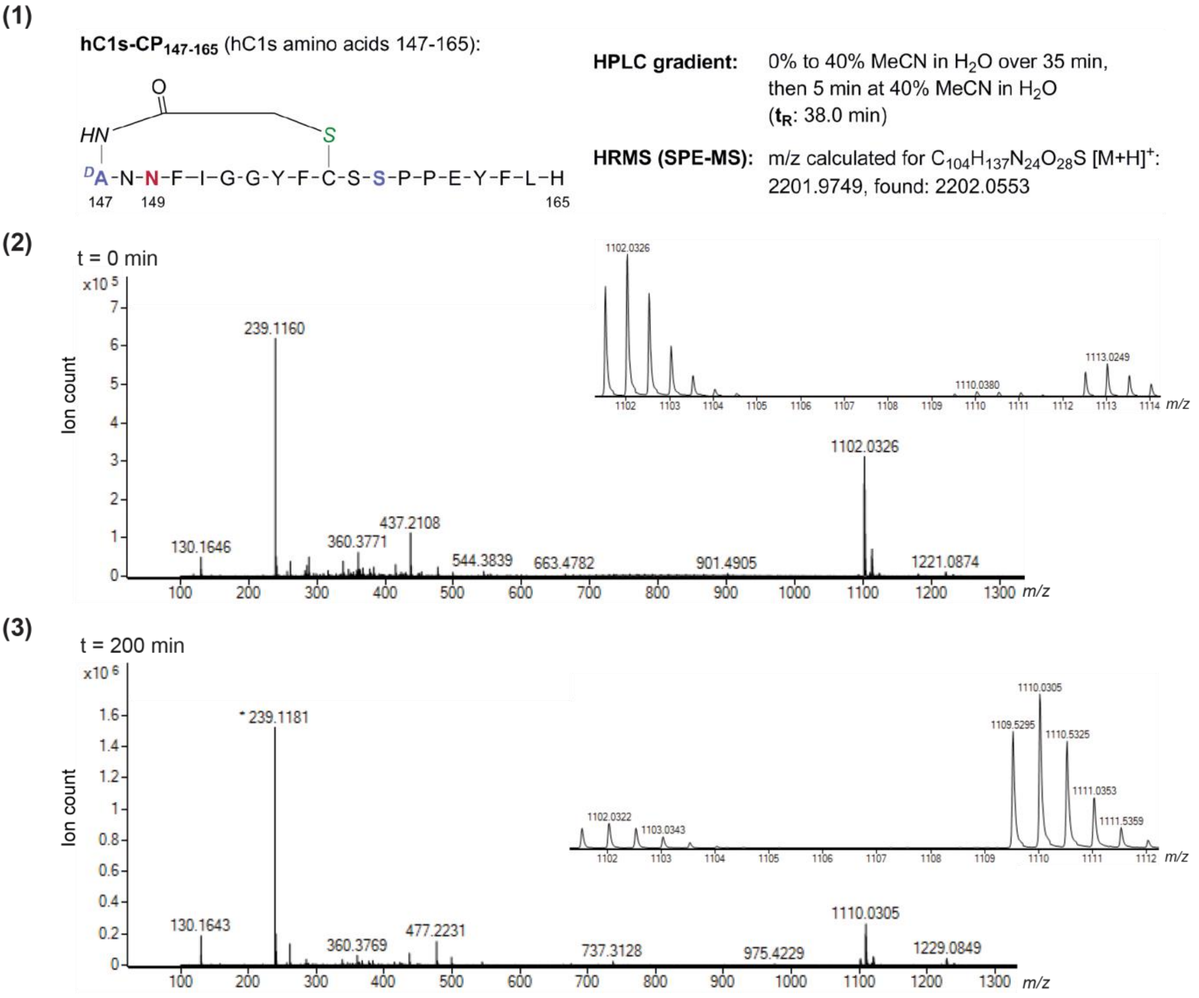

(h) The thioether linked cyclic peptide hEGFL7-CP$_{152\text{-}170}$ is based on the EGFD2 amino acid sequence human epidermal growth factor-like protein 7. 1) Sequence, structure, and analytic characteristics of hEGFL7-CP$_{152\text{-}170}$. 2) Mass spectrum (SPE-MS) of the reaction mixture containing hEGFL7-CP$_{152\text{-}170}$ before the addition of His$_6$-AspH$_{315\text{-}758}$ (t = 0 min). Both HEPES (239.1 Da, m/z +1) and hEGFL7-CP$_{152\text{-}170}$ (1061.5 Da, m/z +2 including sodium ion and HEPES adducts) were observed; the enlarged region shows the m/z +2 peak. 3) Mass spectrum (SPE-MS) of the same reaction mixture 15 min after the addition of His$_6$-AspH$_{315\text{-}758}$; the conversion was >95%. Both HEPES (239.1 Da, m/z +1) and the hydroxylated cyclic peptide hEGFL7-CP$_{152\text{-}170}$(O) (1069.5 Da, m/z +2) were observed; the enlarged region shows the m/z +2 peak.

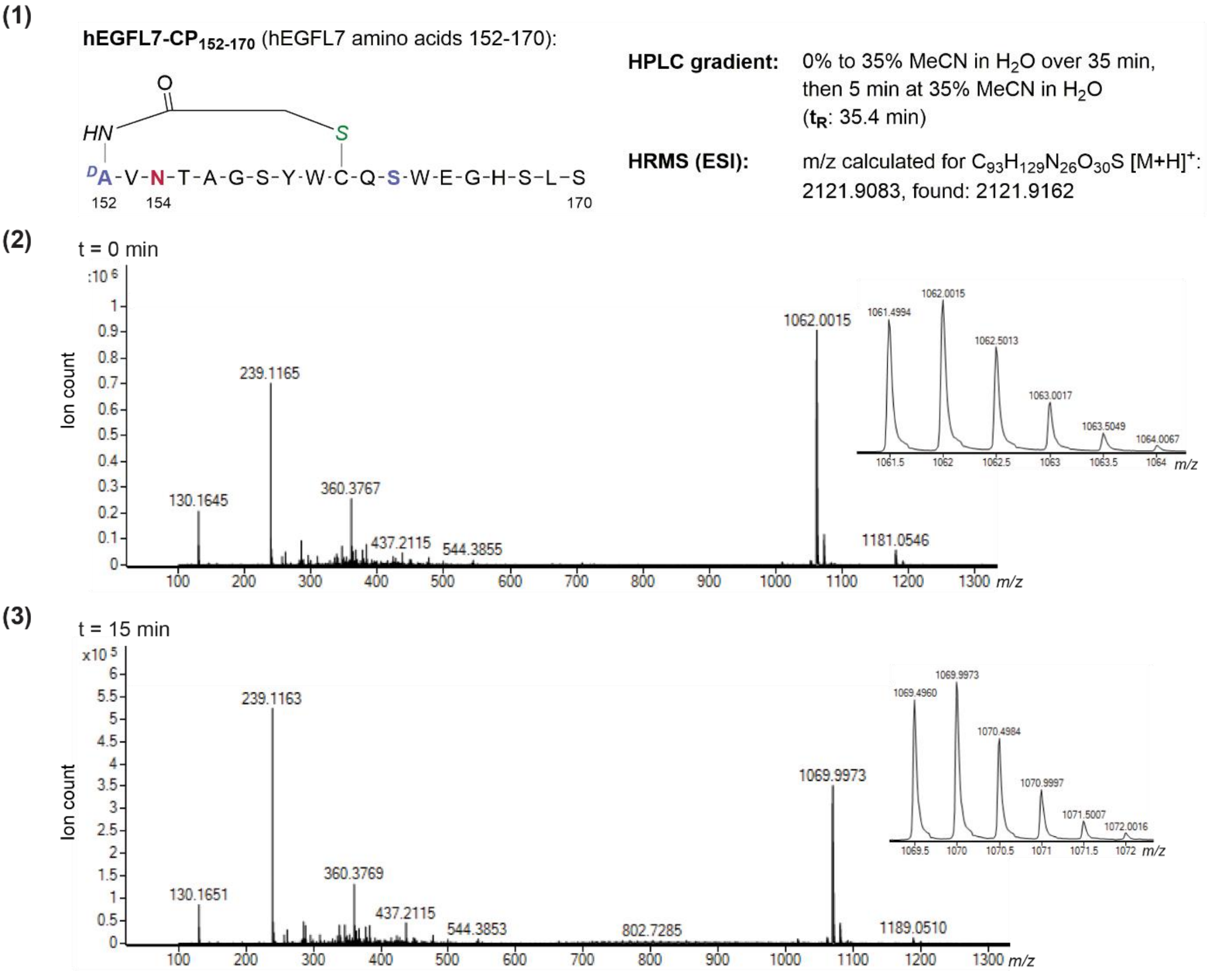

Figure S5. Initial hydroxylation rates of AspH-catalyzed peptide hydroxylation to determine the concentration of active sites. The active site concentration of AspH was determined in independent triplicates using 2,4-pyridinedicarboxylic acid (2,4-PDCA) as a tight binding AspH inhibitor (2,3) using SPE-MS by monitoring the hydroxylation of hFX-CP$_{101-119}$ (4, Figure 1b) in 50 mM HEPES (pH 7.5, 20 °C) as described in the Experimental Procedures. Data are shown as the mean average of three independent runs (n = 3; mean ± standard deviation, SD). (a) Time course of the AspH-catalyzed hydroxylation reaction of hFX-CP$_{101-119}$ for the shown 2,4-PDCA concentrations using 0.1 μM His$_6$-AspH$_{315-758}$, 2.0 μM hFX-CP$_{101-119}$, 100 μM LAA, 2.0 μM FAS, and 3.0 μM 2OG; (b) Initial linear hydroxylation rates used to determine the concentration of AspH active sites. Measurement times were normalized to the first sample injection analyzed after the addition of the Enzyme Mixture to the Substrate Mixture (t = 0 s), by which time low levels of hydroxylation were manifest.

(a)

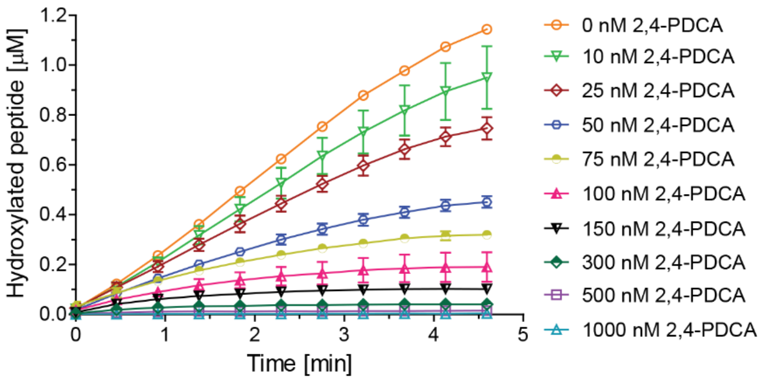

(b)

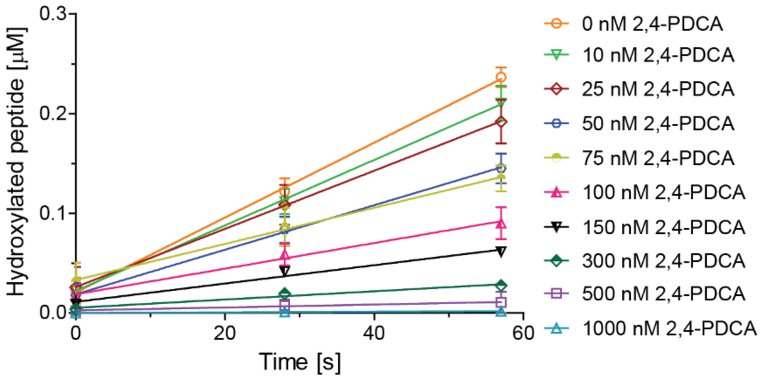

Figure S6. Initial hydroxylation rates of AspH-catalyzed peptide hydroxylations (continues on the following four pages). Maximum velocities (v_{max}^{app}) and Michaelis constants (K_m^{app}) of AspH were determined in independent triplicates for Fe(II), LAA, and 2OG, monitoring (SPE-MS) hydroxylation of hFX-CP$_{101-119}$ (4, Figure 1b) in 50 mM HEPES (pH 7.5, 20 °C) as described in the Experimental Procedures. In a similar fashion, v_{max}- and K_m-values of AspH were determined in independent triplicates for hFX-CP$_{101-119}$, hProC-CP$_{111-129}$, hC1r-CP$_{165-183}$, hEGFL7-CP$_{152-170}$, and hC1s-CP$_{147-165}$. Measurement times were normalized to the first sample injection analyzed after the addition of the Enzyme Mixture to the Substrate Mixture (t = 0 s), by which time low levels of hydroxylation were manifest. Data are shown as the mean average of three independent runs (n = 3; mean ± standard deviation, SD).

(a) Time course of the AspH-catalyzed hydroxylation reaction of hFX-CP$_{101-119}$ (4, Figure 1b) for the shown FAS concentrations using 50 µM 2OG; and (b) initial linear hydroxylation rates used to determine kinetic parameters of AspH for Fe(II) in the absence of LAA.

(a)

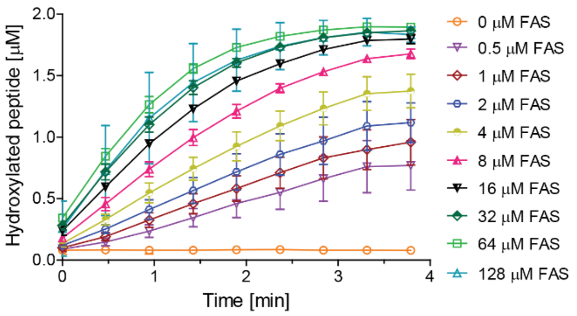

(b)

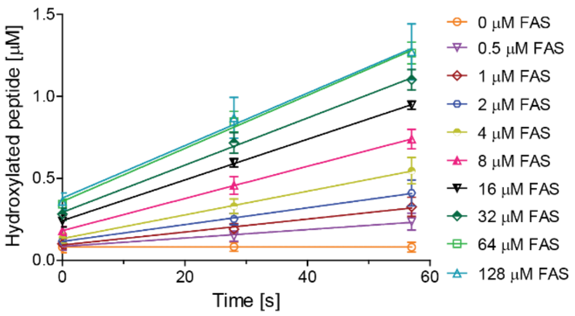

(c) Time course of the AspH-catalyzed hydroxylation reaction of hFX-CP$_{101-119}$ (4, Figure 1b) for the shown FAS concentrations using 100 µM LAA, and 50 µM 2OG; and (d) initial linear hydroxylation rates used to determine kinetic parameters of AspH for Fe(II) in the presence of LAA.

(c)

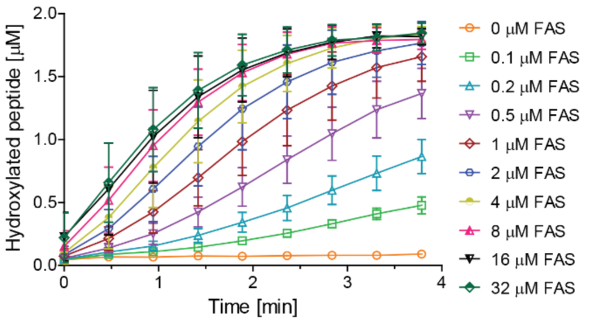

(d)

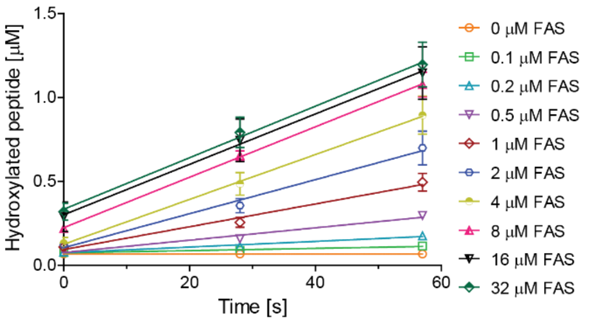

(e) Time course of the AspH-catalyzed hydroxylation reaction of hFX-CP$_{101-119}$ (4, Figure 1b) for the shown LAA concentrations using 50 µM FAS, and 50 µM 2OG; and (f) initial linear hydroxylation rates used to determine kinetic parameters of AspH for LAA.

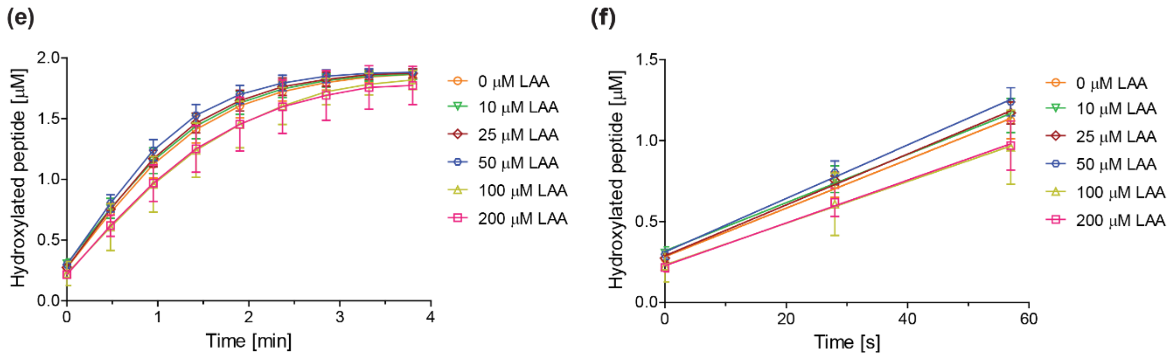

(g) Time course of the AspH-catalyzed hydroxylation reaction of hFX-CP$_{101-119}$ (4, Figure 1b) for the shown 2OG concentrations using 50 µM FAS; and (h) initial linear hydroxylation rates used to determine kinetic parameters of AspH for 2OG.

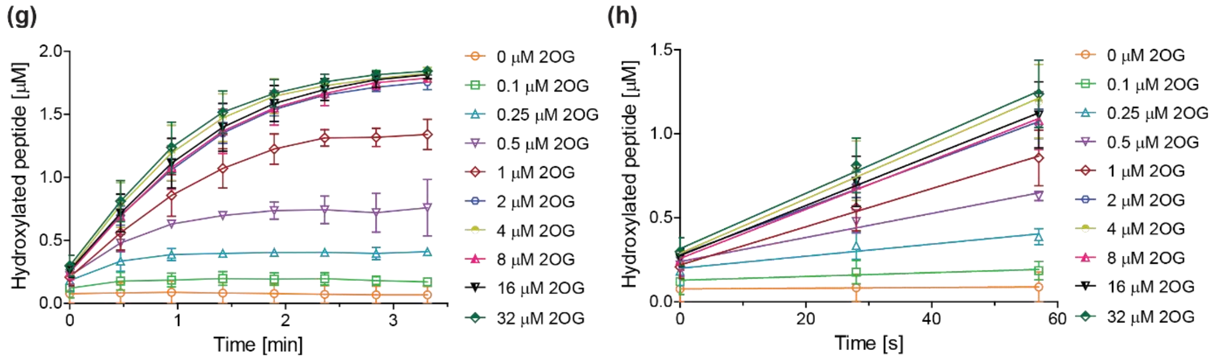

(i) Time course of the AspH-catalyzed hydroxylation reaction of hFX-$CP_{101-119}$ (4, Figure 1b) for the shown hFX-$CP_{101-119}$ concentrations using 100 μM LAA, 20 μM FAS, and 20 μM 2OG; and (j) initial linear hydroxylation rates used to determine kinetic parameters of AspH for hFX-$CP_{101-119}$.

(i)

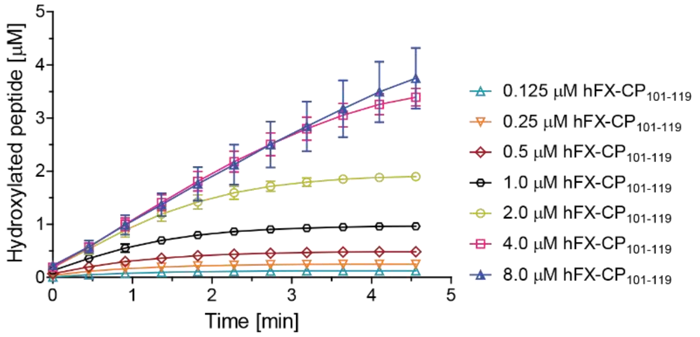

(j)

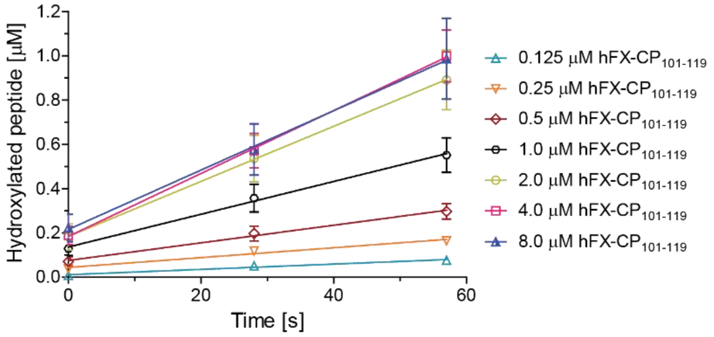

(k) Time course of the AspH-catalyzed hydroxylation reaction of hProC-$CP_{111-129}$ for the shown hProC-$CP_{111-129}$ concentrations using 100 μM LAA, 20 μM FAS, and 20 μM 2OG; and (l) initial linear hydroxylation rates used to determine kinetic parameters of AspH for hProC-$CP_{111-129}$.

(k)

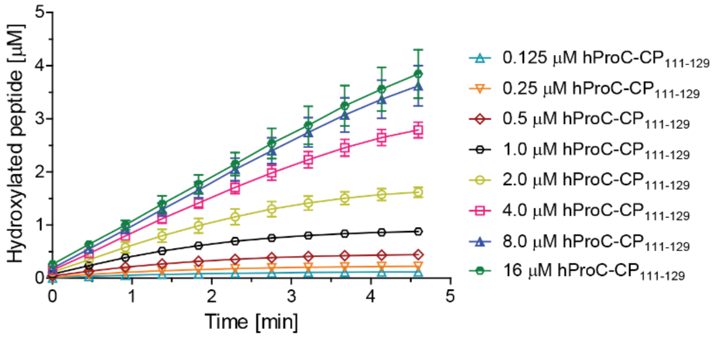

(l)

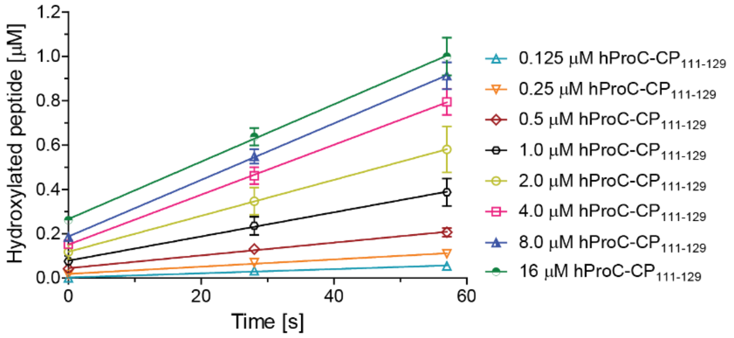

(m) Time course of the AspH-catalyzed hydroxylation reaction of hC1r-CP165-183 for the shown hC1r-CP165-183 concentrations using 100 μM LAA, 20 μM FAS, and 20 μM 2OG; and (n) initial linear hydroxylation rates used to determine kinetic parameters of AspH for hC1r-CP165-183.

(m)

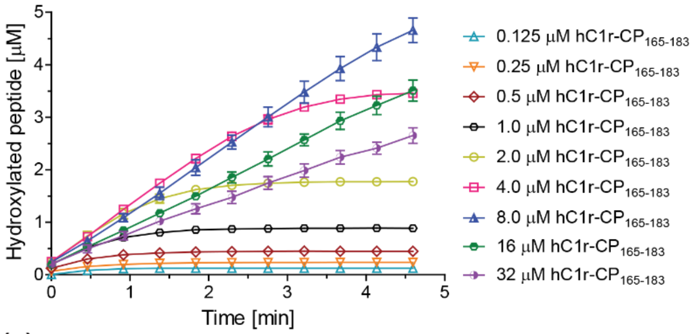

(n)

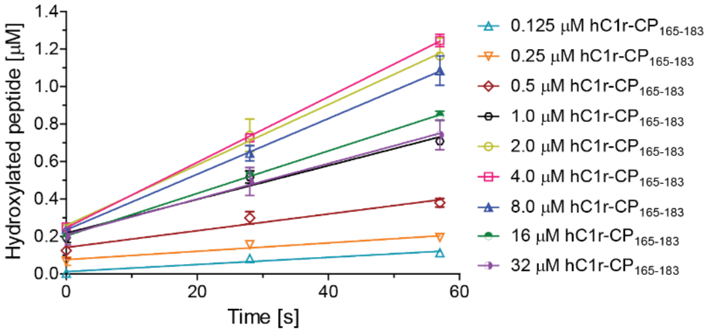

(o) Time course of the AspH-catalyzed hydroxylation reaction of hEGFL7-CP152-170 for the shown hEGFL7-CP152-170 concentrations using 100 μM LAA, 20 μM FAS, and 20 μM 2OG; and (p) initial linear hydroxylation rates used to determine kinetic parameters of AspH for hEGFL7-CP152-170.

(o)

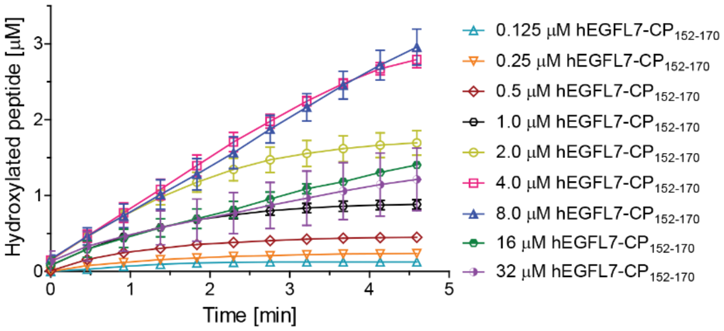

(p)

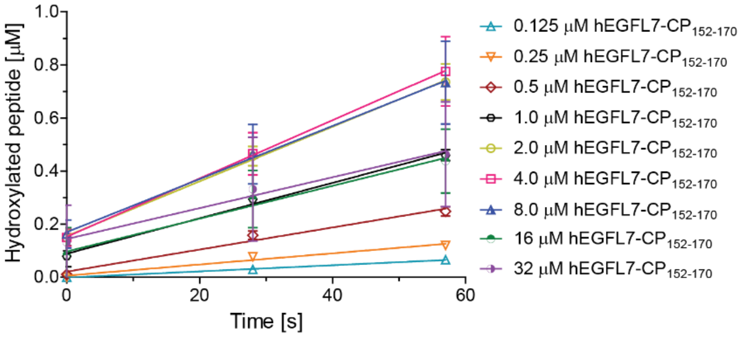

(q) Time course of the AspH-catalyzed hydroxylation reaction of hC1s-CP$_{147\text{-}165}$ for the shown hC1s-CP$_{147\text{-}165}$ concentrations using 100 µM LAA, 20 µM FAS, and 20 µM 2OG; and (r) initial linear hydroxylation rates used to determine kinetic parameters of AspH for hC1s-CP$_{147\text{-}165}$.

(q)

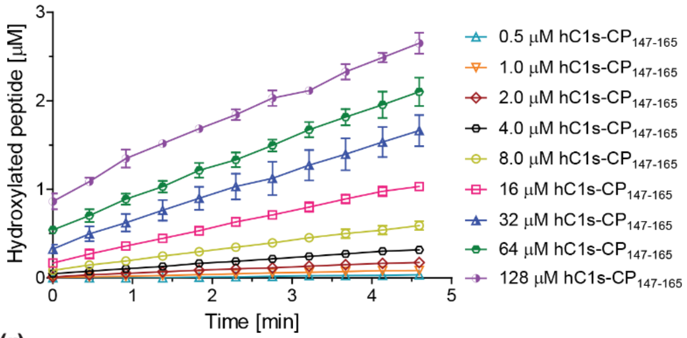

(r)

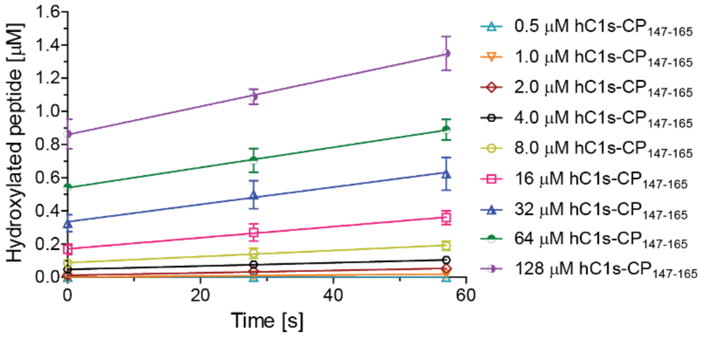

Figure S7. Initial hydroxylation rates of AspH-catalyzed peptide hydroxylations to investigate the O_2 dependence of AspH. Maximum velocity (v_{max}^{app}) and Michaelis constant (K_m^{app}) of AspH were determined for O_2 in independent quadruplicate assays in 50 mM HEPES (pH 7.5, 20 °C) as described in the Experimental Procedures. The O_2 [%] was converted into O_2 [µM] by standard calibration ($y_{[\mu M]} = 9.026 \cdot x_{[\%]}$) (4). Data are shown as the mean average of four independent runs (n = 4; mean ± standard deviation, SD). (a) Initial hydroxylation rates of hFX-$CP_{101-119}$ (4, Figure 1b) for the shown O_2 concentrations using 100 µM LAA, 20 µM 2OG, 20 µM FAS, 2.0 µM hFX-$CP_{101-119}$, and 0.1 µM His_6-$AspH_{315-758}$; (b) Determination of K_m^{app} of AspH for O_2 [%] (K_m^{app} = 47.2 ± 8.1% O_2).

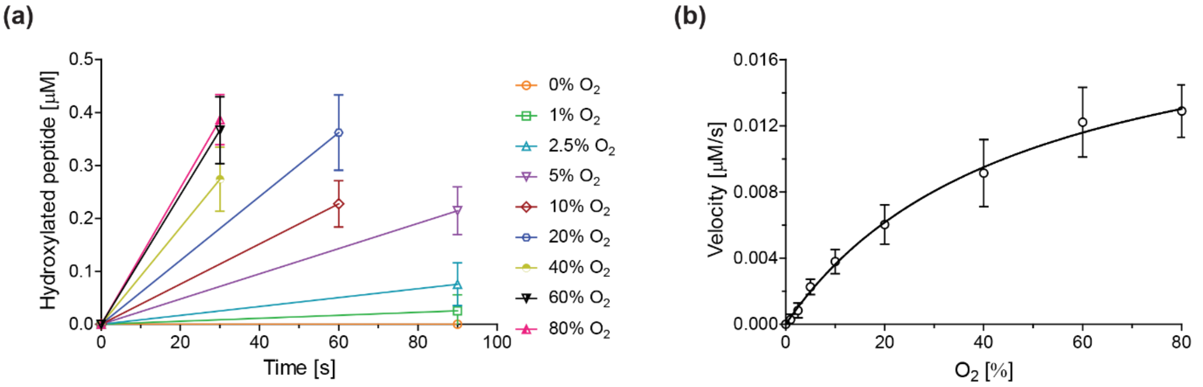

Supporting References

1. Pfeffer, I., Brewitz, L., Krojer, T., Jensen, S. A., Kochan, G. T., Kershaw, N. J., Hewitson, K. S., McNeill, L. A., Kramer, H., Münzel, M., Hopkinson, R. J., Oppermann, U., Handford, P. A., McDonough, M. A., and Schofield, C. J. (2019) Aspartate/asparagine-β-hydroxylase crystal structures reveal an unexpected epidermal growth factor-like domain substrate disulfide pattern. *Nat. Commun.* **10**, 4910

2. Gronke, R. S., Welsch, D. J., VanDusen, W. J., Garsky, V. M., Sardana, M. K., Stern, A. M., and Friedman, P. A. (1990) Partial purification and characterization of bovine liver aspartyl β-hydroxylase. *J. Biol. Chem.* **265**, 8558-8565

3. Derian, C. K., VanDusen, W., Przysiecki, C. T., Walsh, P. N., Berkner, K. L., Kaufman, R. J., and Friedman, P. A. (1989) Inhibitors of 2-ketoglutarate-dependent dioxygenases block aspartyl β-hydroxylation of recombinant human factor IX in several mammalian expression systems. *J. Biol. Chem.* **264**, 6615-6618

4. Hancock, R. L., Masson, N., Dunne, K., Flashman, E., and Kawamura, A. (2017) The activity of JmjC histone lysine demethylase KDM4A is highly sensitive to oxygen concentrations. *ACS Chem. Biol.* **12**, 1011-1019
